# Bio-fertilizer Affects Structural Dynamics, Function, and Network Patterns of the Sugarcane Rhizospheric Microbiota

**DOI:** 10.1007/s00248-021-01932-3

**Published:** 2021-11-24

**Authors:** Qiang Liu, Ziqin Pang, Zuli Yang, Fallah Nyumah, Chaohua Hu, Wenxiong Lin, Zhaonian Yuan

**Affiliations:** 1 Key Laboratory of Sugarcane Biology and Genetic Breeding, Ministry of Agriculture, Fujian Agriculture and Forestry University, Fuzhou, 350002 China; 2 College of Agricultural, Fujian Agriculture and Forestry University, Fuzhou, 350002 China; 3 Fujian Provincial Key Laboratory of Agro-Ecological Processing and Safety Monitoring, College of Life Sciences, Fujian Agriculture and Forestry University, Fuzhou, 350002 China; 4 Key Laboratory of Crop Ecology and Molecular Physiology, Fujian Agriculture and Forestry University, Fuzhou, 35002 China; 5 Province and Ministry Co-Sponsored Collaborative Innovation Center of Sugar Industry, Nanning, 530000 China; 6 Guangxi Laibin Xinbin Commercial Crop Technology Extension Station, Laibin, 546100 Guangxi China

**Keywords:** Bio-fertilizer, Physicochemical property, Rhizosphere microbes, SVM, Sugarcane

## Abstract

Fertilizers and microbial communities that determine fertilizer efficiency are key to sustainable agricultural development. Sugarcane is an important sugar cash crop in China, and using bio-fertilizers is important for the sustainable development of China’s sugar industry. However, information on the effects of bio-fertilizers on sugarcane soil microbiota has rarely been studied. In this study, the effects of bio-fertilizer application on rhizosphere soil physicochemical indicators, microbial community composition, function, and network patterns of sugarcane were discussed using a high-throughput sequencing approach. The experimental design is as follows: CK: urea application (57 kg/ha), CF: compound fertilizer (450 kg/ha), BF1: bio-fertilizer (1500 kg/ha of bio-fertilizer + 57 kg/ha of urea), and BF2: bio-fertilizer (2250 kg/ha of bio-fertilizer + 57 kg/ha of urea). The results showed that the bio-fertilizer was effective in increasing sugarcane yield by 3–12% compared to the CF treatment group, while reducing soil acidification, changing the diversity of fungi and bacteria, and greatly altering the composition and structure of the inter-root microbial community. Variance partitioning canonical correspondence (VPA) analysis showed that soil physicochemical variables explained 80.09% and 73.31% of the variation in bacteria and fungi, respectively. Redundancy analysis and correlation heatmap showed that soil pH, total nitrogen, and available potassium were the main factors influencing bacterial community composition, while total soil phosphorus, available phosphorus, pH, and available nitrogen were the main drivers of fungal communities. Volcano plots showed that using bio-fertilizers contributed to the accumulation of more beneficial bacteria in the sugarcane rhizosphere level and the decline of pathogenic bacteria (e.g., *Leifsonia*), which may slow down or suppress the occurrence of diseases. Linear discriminant analysis (LDA) and effect size analysis (LEfSe) searched for biomarkers under different fertilizer treatments. Meanwhile, support vector machine (SVM) assessed the importance of the microbial genera contributing to the variability between fertilizers, of interest were the bacteria *Anaerolineace*, *Vulgatibacter*, and *Paenibacillus* and the fungi *Cochliobolus*, *Sordariales*, and *Dothideomycetes* between CF and BF2, compared to the other genera contributing to the variability. Network analysis (co-occurrence network) showed that the network structure of bio-fertilizers was closer to the network characteristics of healthy soils, indicating that bio-fertilizers can improve soil health to some extent, and therefore if bio-fertilizers can be used as an alternative to chemical fertilizers in the future alternative, it is important to achieve green soil development and improve the climate.

**Supplementary Information:**

The online version contains supplementary material available at 10.1007/s00248-021-01932-3.

## Introduction

Increasing population numbers are putting tremendous pressure and challenges on global food demand and land productivity [1, 2]. Soil fertility degradation has been a key agricultural concern [3, 4]. Overuse of chemical fertilizers in some growing agricultural areas, especially over-reliance on nitrogen fertilizers, has led to an imbalance in the nutrient structure of fertilizer supply and a decrease in fertilizer utilization [5, 6]. Such unreasonable agronomic measures lead to soil nutrient imbalance, gradual decline of crop growth, reduction of the content of soil organic matter, destruction of soil agglomeration structure, and a reduction of the activity of soil microorganisms that are closely related to plant growth [7–9]. In addition, intensive agricultural practices characterized by using high levels of chemical fertilizers and pesticides can alter soil biology by disrupting biological interactions. Such measures may lead to the rapid development of soil-borne diseases with imbalances in the subsurface microbiosphere caused by the proliferation of harmful soil microorganisms, including plant pathogenic fungi and bacteria. Therefore, in this context, the development of new bio-fertilizers will bring a fresh turn in agricultural production. Modern agriculture has increasingly focused on the use of bio-fertilizer as alternatives to chemical fertilizers. Numerous studies have shown that the application of bio-fertilizers can inhibit the development of related soil-borne diseases by reshaping the plant rhizosphere microbiota and promoting the secretion of related chemicals such as carbohydrates, amino acids, organic acids, proteins, and enzymes [10, 11]. Indoor cultivation trials by Dong et al. showed that soil and microorganisms under bio-fertilizer treatment conditions were significantly more resistant to pathogenic bacteria than those treated with chemical fertilizers after a spiking of *Ralstonia solanacearum* [12]. The study by Zhang et al. also showed that using *Trichoderma* bio-fertilizer can increase soil antifungal compounds, and it was speculated that it may suppress pathogenic bacteria and be an important reason for increasing grass biomass [13]. It has also been shown that the application of bio-fertilizers improves soil organic matter content, pH, soil microbial activity, and diversity more than the application of chemical fertilizers alone [14]. Most of these studies have focused on model crops or indoor cultivation conditions, and the response of rhizosphere microorganisms to bio-fertilizer under real production and field conditions remains elusive.

Soil is a very complex ecosystem in which different microorganisms play different roles [15, 16]. Plants have been placed in a sea of microorganisms from the time they were planted. Mechanisms of growth evolution have led plants to know how to find partner microorganisms that work together below adversity [17]. Plant growth-promoting bacteria (PGPB) and plant growth-promoting fungi (PGPF) can work hand in hand with plants [18]. Meanwhile, soil microbes are sensitive to environmental stresses and they play an important role in fertilizer nutrient conversion. The importance of rhizosphere microbes as neighbors of plant roots for plant health and growth cannot be overstated [15, 20]. Rhizosphere microbial communities can promote the growth of plant above-ground tissues by enhancing adaptation to environmental stresses, improving nutrient acquisition, and improving plant metabolic functions. A study by Singh et al. demonstrated the defense response of a rhizosphere microbial community consisting of *Pseudomonas* (PHU094), *Trichoderma* (THU0816), and *Rhizobium* (RL091) strains to specific biotic stresses in chickpea [21]. In addition, Yi et al. showed that plants can defend themselves against herbivore attack by self-protection mechanisms that recruit beneficial microorganisms of plant-promoting rhizobacteria/fungi [22]. Furthermore, Solanki et al. published that in intercropping systems, abundant plant rhizosphere beneficial diazotrophs can promote plant growth and act as an effective biological inoculant to sustain sugarcane production, and this exploration of rhizosphere microbes can provide an excellent solution to reduce the overuse of chemical fertilizers [5]. Breakthroughs in the study of rhizosphere microbial communities will open the door to microbial regulation of plant growth and metabolism. With the increasing exploration of soil microbial potential and the deepening of the concept of sustainable development, green and healthy bio-fertilizer will become the preferred choice for agricultural production. The objectives of our study were (a) to investigate the relationship between changes in the rhizosphere microbial community of sugarcane and different fertilizer application regimes and to reveal the correlation between soil microbial composition and soil chemical properties, (b) to determine the network characteristics of microorganisms under different fertilizers, and (c) to determine the contribution of bio-fertilizer application to sustainable agriculture.

## Materials and Methods

### Plant Materials and Fertilizers

FN41 sugarcane variety was obtained from the sugarcane experiment site of Fujian Agriculture and Forestry University. Chemical fertilizer was bought from Meishan Xindu Chemical Compound Fertilizer Co., Ltd., and its total nutrient (N-P_2_O_5_-K_2_O: 15–15-15) ≥ 45%. The bio-fertilizer is a compound microbial fertilizer provided by Jiangyin Lianye Biology Co., Ltd., which is developed by Nanjing Agricultural University. Bio-fertilizer was produced by inoculation of *Bacillus* amyloliquefaciens T-5 [23] into a mixture of rapeseed meal and chicken manure composts for the solid fermentation process. The properties of the bio-fertilizer were (N + P_2_O_5_ + K_2_O) = 8%, effective living bacteria ≥ 20 million/g, and organic matter ≥ 20%. The fertilizer application calculation tool (version 1.1) for the experimental plots was used to determine the amount of fertilizer to be applied.

### Experimental Description and Soil Samples

A field experiment was conducted at the Sugarcane Research Station in Xingbin District, Guangxi Province of China, from March 7, 2017 to December 20, 2017. The climate is mainly subtropical monsoon climate. The annual average temperature and annual precipitation are located in the range of 20–22℃ and 1300–1350 mm, respectively. The pre-test soil samples were collected on March 1, 2017, stored on ice, and transported back to the laboratory where the determination of physicochemical properties began immediately, and the physicochemical properties were as follows: pH (4.82), soil organic matter (SOC, 17.50 g ·kg^–1^), total nitrogen (TN, 1.29 g· kg^–1^), available potassium (AK, 54.16 mg· kg^–1^), and available phosphorus (AP, 45.19 mg· kg^–1^). The treatments are as follows: (1) CK: urea application (57 kg/ha), CF: compound fertilizer (450 kg/ha), BF1: bio-fertilizer (1500 kg/ha of bio-fertilizer + 57 kg/ha of urea), and BF2: bio-fertilizer (2250 kg/ha of bio-fertilizer + 57 kg/ha of urea). Fertilizer was applied at different periods, the first application was made at the seedling stage (March 10, 2017), accounting for 40% of the total fertilizer application, and the second was made at the elongation stage (July 10, 2017), accounting for 60%. Each plot contained 5 rows. The field experiment was conducted in a randomized block design, and the row spacing was 1.2 m and row length was 25 m. Sugarcane yields and sugar content were evaluated and soil samples were collected during the maturity period. Nine soil cores from one field plot were pooled into one sample [24], and a total of 12 field plot samples were collected, including four fertilization treatments × three replications. All samples were placed individually in sterile bags and sent to the laboratory, and stored at − 20 °C; after each sample collection, the tools used were disinfected with an alcohol wipe. The samples were sieved using a 2-mm mesh, thoroughly homogenized, and divided into two parts. Portion was stored at 4 °C, and then a sufficient amount of soil was taken out and dried naturally for the determination of soil physical and chemical properties, while the other portion was stored at − 20 °C for DNA extraction.

### Determination of Soil Physicochemical and Sugarcane Yield Indicators

Soil pH was estimated with a glass electrode using a soil-to-water ratio of 1:2.5, and the soil total nitrogen (TN) in the extract was determined by Element Analyzer (Thermo Scientific™, Waltham, MA, USA). Soil available phosphorus (AP) was extracted with sodium bicarbonate and determined by the molybdenum blue method. The available nitrogen (AN) and available potassium (AK) were determined by the alkaline hydrolysis diffusion method and the flame photometric method. In addition, the soil organic carbon content (SOC) was determined by using 0.8 mol/L K_2_Cr_2_O_7_ redox titration method. All soil physical–chemical properties were determined according to Bao [25]. The stem height and diameter of sugarcane were measured by randomly selecting 30 sugarcane plants in each plot and using tape and Vernier caliper. The number of effective stems was extrapolated from the number of effective stems in the area of 1.2 × 2.5 m to the total area of effective stems of sugarcane. To measure the sucrose content, an Extech Portable Sucrose Brix Refractometer (Mid-State Instruments, San Luis Obispo, CA, USA) was used, and the calculation was performed using the following formula: sucrose (%) = Brix (%) × 1.0825 − 7.703 [26]. The theoretical yield of sugarcane was estimated using the following equation:

$$(a) Single stalk weight (kg)\hspace{0.17em}=\hspace{0.17em}(stalk diameter {(cm))}^{2}\hspace{0.17em}\times \hspace{0.17em}(stalk height (cm)\hspace{0.17em}-\hspace{0.17em}30)\hspace{0.17em}\times \hspace{0.17em}1 (g/{cm}^{3})\hspace{0.17em}\times \hspace{0.17em}0.7854/1000$$

$$(b) Theoretical production (kg/{hm}^{2})\hspace{0.17em}=\hspace{0.17em}single stalk weight (kg)\hspace{0.17em}\times \hspace{0.17em}productive stem numbers ({hm}^{2})$$

### Soil DNA Extraction, PCR Amplification, and Sequencing

Deoxyribonucleic acid was extracted from the experimental soil using the Power Soil DNA Isolation Kit (MoBio Laboratories Inc., Carlsbad, USA) according to the manufacturer’s instructions. The quantity and quality of deoxyribonucleic acid (DNA) extracts were analyzed using a NanoDrop 2000 spectrophotometer (Thermo Scientific, Waltham, MA, USA) and the DNA was stored at − 80℃ for future analysis [12]. 16S rRNA and 18S rRNA gene fragments were amplified using primers 338F (5′-ACTCCTACGGGAGGCAGCAG-3′)/806R (5′-GGACTACHVGGGTWTCTAAT-3′) [27] and SSU0817F (5′-TTAGCATGGAATAATRRAATAGGA-3′)/SSU1196R (5′-TCTGGACCTGGTGAGTTTCC-3′) [28], respectively. The amplification condition was 95℃ for 3 min, followed by 35 cycles of 95℃ for 30 s, 55℃ for 30 s, and 72℃ for 45 s, with a final extension at 72℃ for 10 min (GeneAmp 9700, ABI, California CA, USA). PCR reaction was performed in triplicate in a 20-μL mixture containing 2 μL of 2.5 mM deoxyribonucleoside triphosphate (dNTPs), 4 μL of 5 × Fast Pfu buffer, 0.4 μL of Fast Pfu polymerase, 0.4 μL of each primer (5 μM), and template DNA(10 ng) [29]. Extraction of amplicons was carried out using an AxyPrep DNA Gel Extraction Kit (Axygen Biosciences, Union City, CA, USA). Then, QuantiFluor™-ST (Promega, Madison, WI, USA) was used for quantification. Purified amplicons were pooled in equimolar and paired-end sequenced (2 × 250) on an Illumina MiSeq platform (Majorbio, Shanghai) according to the standard protocols. The UPARSE standard pipeline was used to analyze the sequence data [30]. Briefly, sequences with short reads (< 250 bp) were filtered out prior to downstream analysis [31]. Sequences with ≥ 97% similarity were clustered into OTUs, and the taxonomic assignment was performed using the RDP database (http://rdp.cme.msu.edu/). All sequences were deposited in the NCBI Sequence Read Archive database with the accession number PRJNA682545.

### Statistical Analysis

For subsequent analyses, minimum numbers of sequences were extracted at random from each sample to calculate an alpha diversity index. The significance of soil nutrients and sugarcane yield indicators was calculated using DPS, based on the LSD test (*P* < 0.05). Box plots of α-diversity indices, species composition, Venn diagrams, and correlation heatmap (Spearman correlation) were performed using R (3.5.2). The difference analysis (DESeq2) and VPA (variance partitioning canonical correspondence) analysis were also calculated and visualized using R [32, 33]. Bray–Curtis distance was calculated by “vegdist” function of vegan package on R (3.4.0). Non-parametric multivariate analysis of variance (PERMANOVA) was performed with the Adonis function in the vegan package of R based on the Bray–Curtis distance. Support vector machine (SVM) analysis is as follows: first logarithmic transformation of relative abundance data, then intra-matrix correction of data, and finally using Wekemo biointiomnatios cloud platform (https://bioincloud.tech) to complete [34]. Co-occurrence networks were done using the R (version 4.0.3) and Cytoscape software (3.6.1), and network structure analysis was done using UCINET (version 6.186) to calculate mean degree, clustering coefficient, and other parameters [35]. Bacterial functions were predicted by the PICRUSt software based on the KEGG functional database and fungi were annotated using the FUNGuild database [36, 37].

## Results

### Sugarcane Yield Index and Soil Nutrient Variability

Compared to CF treatment, the yield per hectare of FN41 sugarcane increased from 3 to 12% under the bio-fertilizer amendment soil (BF1 and BF2). Furthermore, compared to CK, BF1, BF2, and CF treatments significantly increased (*P* < 0.05) plant height, stem weight, and effective stem. However, sugarcane stem diameter under CF, BF1, and BF2 treatments revealed no significant difference compared to CK treatment (Table 1). Compared with CK and CF treatments, soil pH was significantly higher (*P* < 0.05) in both BF1 and BF2 treatments. However, CF treatment significantly reduced soil pH compared with CK. Moreover, soil organic carbon and available phosphorus were not impacted in all the treatments compared to CK treatment. Compared to CK treatment, soil total nitrogen was significantly higher (*P* < 0.05) in both BF1 and BF2 treatments, whereas soil available nitrogen did not change considerably among all the treatments. The contents of total nitrogen, available nitrogen, total phosphorus, and available potassium increased significantly by about 13.8–33.8%, 12.6–25.0%, 43.8–56.3%, and 97.4–169.5%, respectively, with the increase in BF1 treatment group being the most significant (Table 2).

**Table 1:** Effects of different treatments on yield indexes of sugarcane

Treatment	Yield index					
	Sugar (%)	Plant height (cm)	Stem diameter (cm)	Single stem weight (kg)	Effective stem/ha	Yield/hm^2^
CK	14.17 ± 0.20^ab^	270.05 ± 3.74^c^	2.56 ± 0.06^a^	1.24 ± 0.06^b^	4068 ± 127^b^	75,531 ± 4736^b^
CF	13.43 ± 0.17^b^	286.37 ± 8.10^b^	2.78 ± 0.04^a^	1.57 ± 0.07^a^	4569 ± 113^a^	107,431 ± 6172^a^
BF1	14.32 ± 0.17^a^	303.83 ± 1.82^a^	2.77 ± 0.14^a^	1.66 ± 0.12^a^	4867 ± 212^a^	120,802 ± 12,526^a^
BF2	13.82 ± 0.28^ab^	300.99 ± 2.92^ab^	2.72 ± 0.08^a^	1.59 ± 0.08^a^	4668 ± 96^a^	111,026 ± 7650^a^

Different letters in each column indicate significant differences among the treatments at the 0.05 level

**Table 2:** Effects of different treatments on soil nutrient content of sugarcane

Treatments	Soil chemical properties						
	pH value	Soil organic carbon/(g ·kg^–1^)	Total nitrogen/(g ·kg^−1^)	Total phosphorus/(g· kg^−1^)	Available nitrogen/(mg·kg^−1^)	Available potassium/(mg·kg^−1^)	Available phosphorus/(mg·kg^−1^)
CK	4.93 ± 0.05^b^	17.70 ± 2.69^a^	1.30 ± 0.07^b^	0.32 ± 0.03^b^	90.73 ± 7.32^b^	54.23 ± 6.63^b^	45.26 ± 6.13^a^
CF	4.59 ± 0.07^c^	20.75 ± 1.85^a^	1.48 ± 0.05^ab^	0.46 ± 0.03^a^	102.17 ± 3.49^ab^	107.04 ± 13.66^a^	49.71 ± 3.66^a^
BF1	5.43 ± 0.11^a^	24.48 ± 2.79^a^	1.74 ± 0.18^a^	0.46 ± 0.06^a^	113.44 ± 8.09^a^	146.15 ± 12.94^a^	48.81 ± 9.89^a^
BF2	5.35 ± 0.06^a^	24.81 ± 2.88^a^	1.72 ± 0.10^a^	0.50 ± 0.03^a^	105.99 ± 2.99^ab^	128.37 ± 21.23^a^	45.62 ± 1.68^a^

Different letters indicate a significant difference among treatments based on the LSD test (*p* < 0.05)

### Effect of Different Fertilizers on Rhizosphere Microbial Community and Diversity

**Fig. 1:**
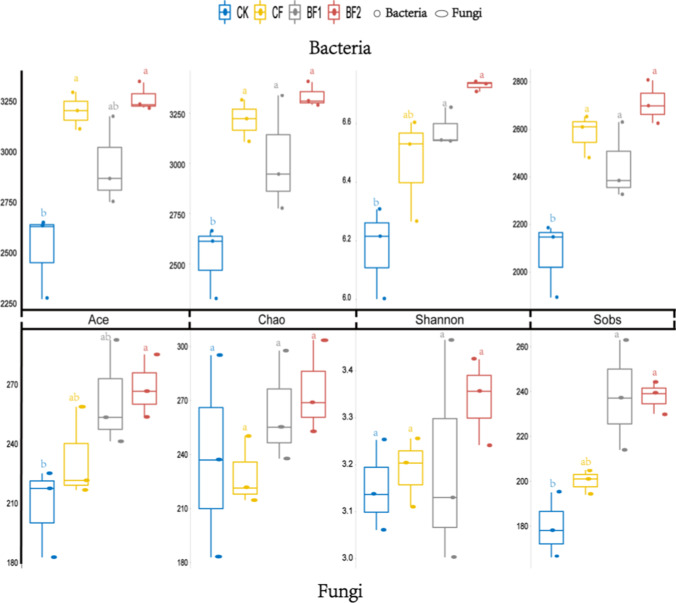
Box plots of rhizosphere microbial alpha diversity index under different fertilizer treatments, Tukey method. CK: urea application (57 kg/ha), CF: compound fertilizer (450 kg/ha), BF1: bio-fertilizer (1500 kg/ha of bio-fertilizer + 57 kg/ha of urea), BF2: bio-fertilizer (2250 kg/ha of bio-fertilizer + 57 kg/ha of urea)

In order to assess the effects of different treatments on microbial alpha diversity in sugarcane rhizosphere soil, we plotted the box-line diagrams (Fig. 1). The rarefaction curve showed the richness of observed OTU, which proved that the depth of sample sequencing was enough to show microbial alpha diversity (Fig. S1). According to the result, rhizosphere bacterial α-diversity (Shannon, Sobs, Chao, and Ace) indices were significantly (*P* ≤ 0.05) affected by fertilizer, but there were differences in the degree of influence between fungi and bacteria (Table S1). For the bacteria, treatments BF1 and BF2 produced the highest significant Shannon indices respectively, compared with CK and CF, and the highest Sobs, Ace, and Chao indices were recorded in treatment BF2 (Table S1). On the other hand, of the fungi, except for Shannon and Chao which were not significantly affected by fertilizer treatment, treatment BF2 registered the highest Sobs and Ace indices compared with other treatments (Table S1).

**Fig. 2:**
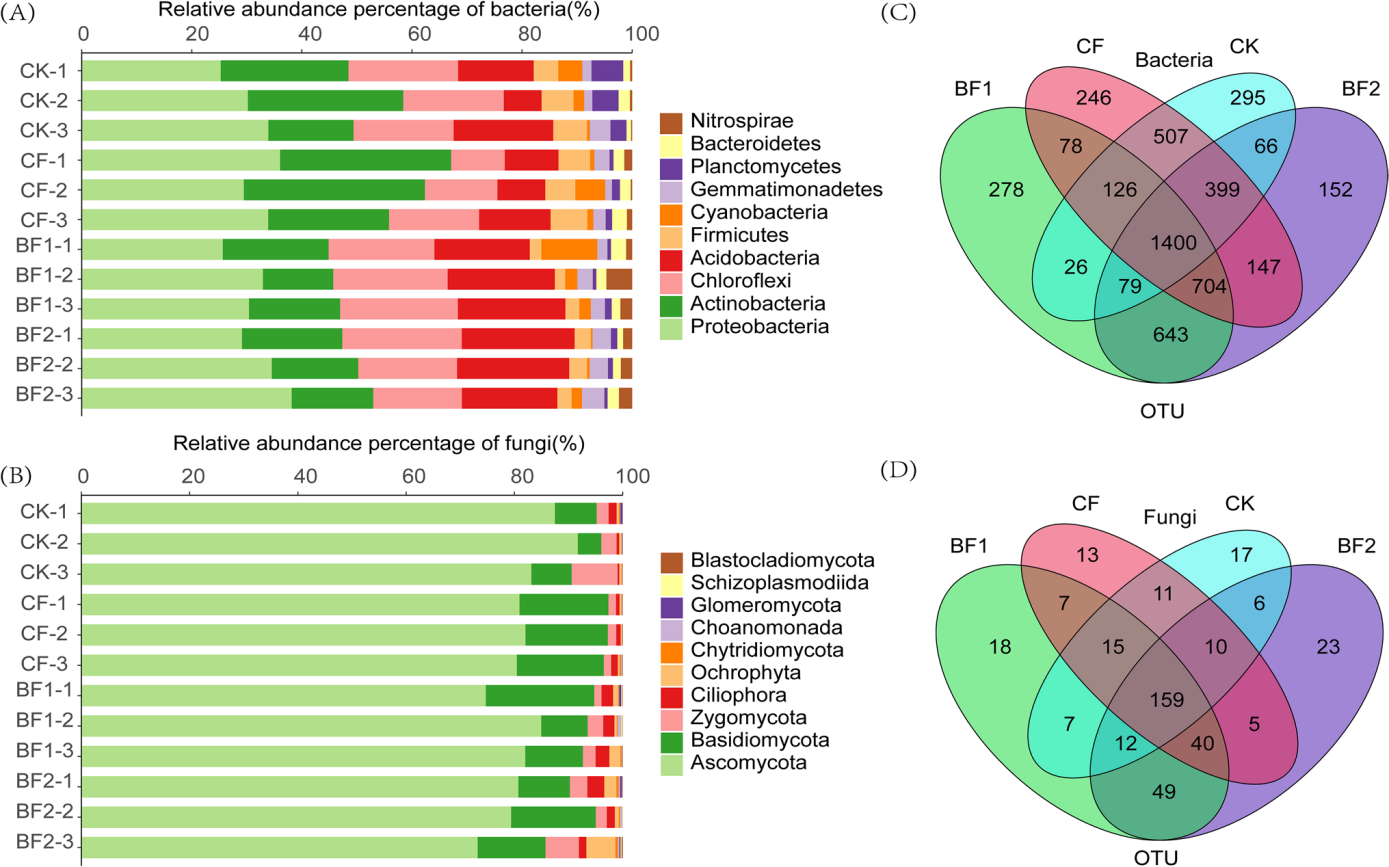
Relative abundance histograms of the top 10 rhizosphere microbial phyla in each sample (**A** and **B**). Comparison of bacterial and fungal OTU using Venn diagram among different fertilizer treatments (**C** and **D**)

The dominant bacteria phyla were Actinobacteria, Proteobacteria, Acidobacteria, Cyanobacteria, Firmicutes, Planctomycetes Bacteroidetes, Chloroflexi, Gemmatimonadetes, and Nitrospirae in all fertilizer treatment soils (Fig. 2A), and the dominant fungi phyla were Ascomycota, Basidiomycota, Zygomycota, Ciliophora, Ochrophyta, Chytridiomycota, Choanomonada, Glomeromycota, Schizoplasmodiida, and Blastocladiomycota (Fig. 2B). Although the dominant phyla of rhizosphere microorganisms in all soils were consistent, changes in the relative abundance of the dominant taxa were observed across different treatments (Table S2). In bacteria, there was a lower abundance of Actinobacteria and a higher abundance of Acidobacteria and Chloroflexi in soils with BF addition comparing with CK and CF (Fig. 2A), and in the OTU level, the addition of fertilizer reduced the number of OTU unique to bacteria in soil, but the degree of decrease was related to the type of fertilizer (Fig. 2C). In addition, Ascomycota had absolute abundance advantage in rhizosphere fungi. Compared to CF, BF treatment has more Ciliophora, Ochrophyta, and Zygomycota (Fig. 2B). In OTU level, the addition of bio-fertilizer makes it have more unique fungal OTU, specifically, CF reduced the number of unique OTUs (Fig. 2D).

**Fig. 3:**
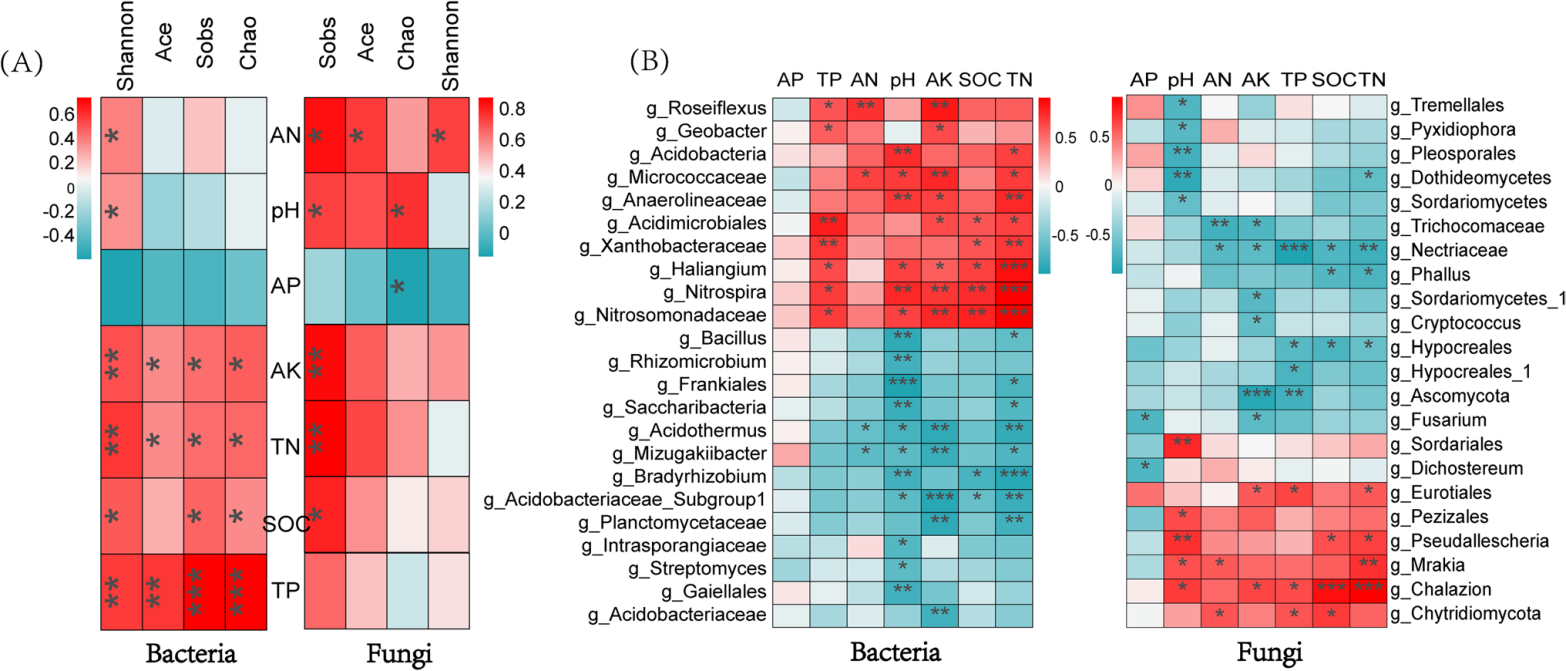
The heatmap of Spearman correlation between microbial alpha diversity index and soil traits (**A**), and a Spearman correlation heatmap of soil environmental variables and the top 30 dominant bacterial and fungal genera, and the correlation coefficient was greater than 0.4, marking the significance level (**B**). * significance at *P* < 0.05, ** significance at *P* < 0.01, and *** significance at *P* < 0.001

The Spearman’s heatmap showed the relationship between microbial diversity and soil traits (Fig. 3A), and the Spearman heatmap correlation analysis between major microbial genera and physiochemical soil variables is also illustrated in Fig. 3B. In bacteria, TP significantly affected the diversity index of bacteria and showed significant positive correlation with Shannon, Ace, Sobs, and Chao (Fig. 3A). In addition, there was a significant correlation between pH, AK, TN, and most of the bacterial genera in bacterial top 30. Among them, genus *Acidobacteria*, *Anaerolineaceae*, and *Nitrospira* had a significant positive correlation with soil pH while *Bacillus*, *Rhizomicrobium*, *Frankiales*, *Saccharibacteria*, and *Bradyrhizobium* were observed to have a significant negative correlation with pH. Furthermore, *Haliangium*, *Nitrospira*, and *Nitrosomonadaceae* had a strongly significant positive correlation with TN, but *Bradyrhizobium* registered a significant negative correlation with TN (Fig. 3B). In fungi, TN and AK had a significant positive correlation with Sobs (Fig. 3A). Meanwhile, *Fusarium* showed a significant negative correlation with AP and AK, and *Ascomycota* also showed a significant negative correlation with TP and AK. It is noteworthy that *Chalazion* showed a significant positive correlation with SOC and TN, and the part of these observations was also confirmed in RDA analysis with the top 10 genera.

**Fig. 4:**
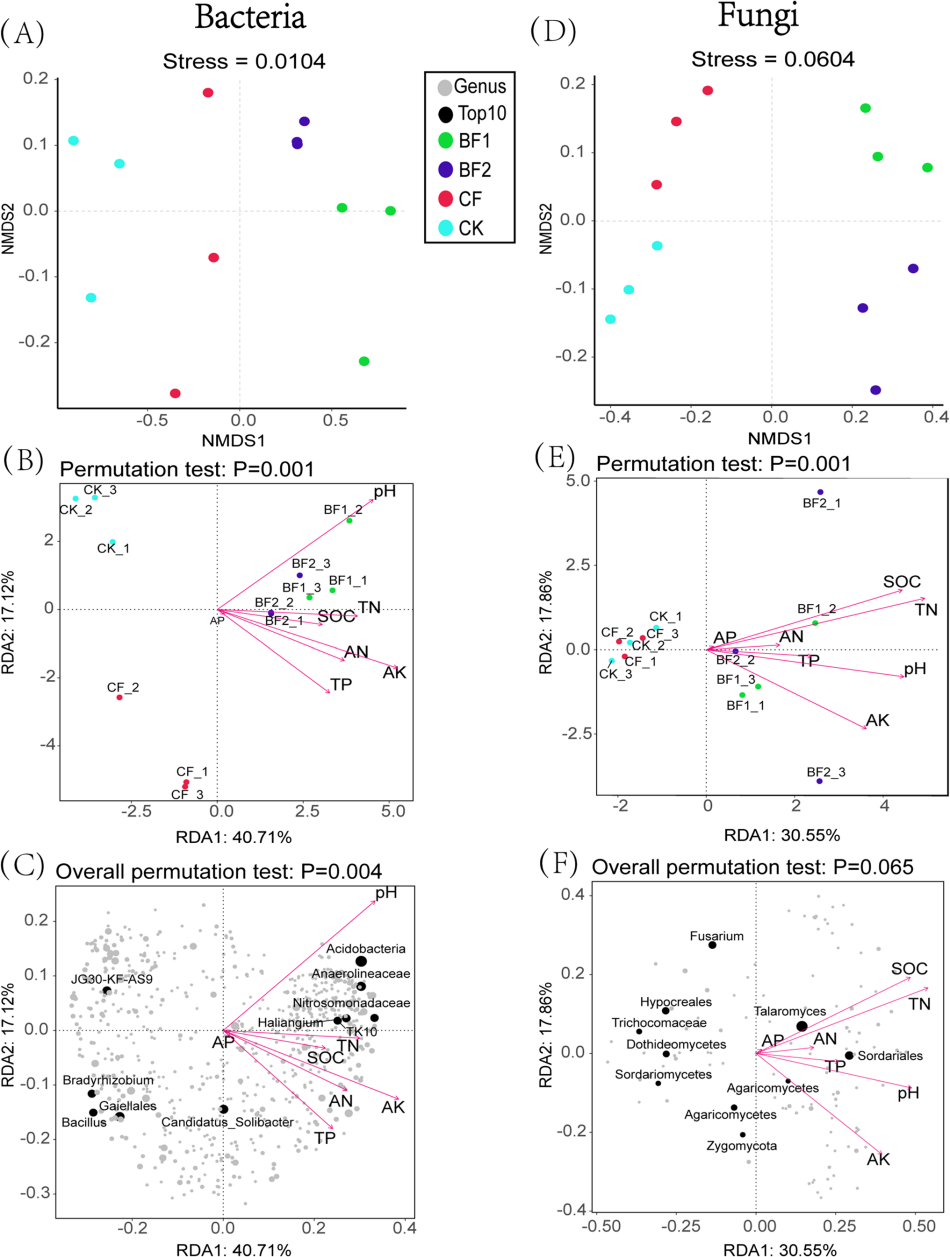
A non-metric multidimensional scaling (NMDS) of rhizosphere microbial community composition among different fertilizer treatments (**A** and **D**). Redundancy analysis (RDA) illustrating association between samples and soil properties among treatments (**B** and **E**), and RDA also indicate association between microbial (top 10 genera) and environmental variables (**C** and **F**). Points with different colors depict sample groups under different fertilizer treatments; gray and black points represent different microbial genera, red arrows represent environmental factors, and the arrow length represents the degree of influence on different genera or samples. Bacteria (**A**-**C**) and fungi (**D**-**F**)

A non-metric multidimensional scaling (NMDS) analysis showed a clear distinction in bacterial and fungal community composition of CK, CF, and BF (Fig. 4A and D). In all the treatments, the bacterial community was distinct from each other based on their NMDS1 axis; however, fungal community composition showed distinct variation among the treatments at their NMDS2 axis. Based on redundancy analysis (RDA), results revealed that soil variables (pH, AN, AK, TN, TP, SOC) affected the soil microbial community in different treatments. The *X* and *Y* canonical axes explained 40.71% and 17.12% and 30.55% and 17.86% of the observed bacterial and fungal species dynamics, respectively. It is worth noting that, of all the soil variables investigated, pH (*r*^2^ = 0.8070, *p*-value = 0.0005) and AK (*r*^2^ = 0.7988, *p*-value = 0.001) in bacteria, SOC (*r*^2^ = 0.6974, *p*-value = 0.0025), TN (*r*^2^ = 0.7558, *p*-value = 0.0020), pH (*r*^2^ = 0.6640, *p*-value = 0.0045), and AK (*r*^2^ = 0.6303, *p*-value = 0.0085) in fungi were observed as important drivers shaping and controlling microbial community (Fig. 4C and F; Table S3). Meanwhile, the results of Adonis test indicated significant differences between different fertilizer treatment groups (Table 3), and VPA analysis showed that soil physicochemical factors explained 80.09% and 73.31% of the variance for bacteria and fungi, respectively, with pH explaining a higher percentage of the variance for fungi (23.88%) than for the bacterial (9.91%) group (Fig. S2).

**Table 3:** Analysis of bacteria and fungi Adonis

	CK		CF		BF1		BF2	
	*R*^2^	*P*	*R*^2^	*P*	*R*^2^	*P*	*R*^2^	*P*
CK			0.593	0.001	0.439	0.002	0.525	0.001
CF	0.447	0.007			0.472	0.004	0.605	0.002
BF1	0.741	0.005	0.654	0.003			0.3	0.005
BF2	0.734	0.004	0.606	0.006	0.478	0.005		

Pairwise comparison of four groups of fertilization measures. The value of *R*^2^ represents the degree of explanation of sample differences, and the higher the value of *R*^2^, the higher the degree of explanation of differences in groups. The left lower triangle represents bacteria, the right upper triangle represents fungi, *R*^2^ > 0.75 is usually interpreted as a clear separation, *R*^2^ > 0.5 indicates separation, and *R*^2^ < 0.25 indicates a group that is difficult to separate. (*p* < 0.05)

### Differential Microorganisms Under Different Fertilizer Treatments

**Fig. 5:**
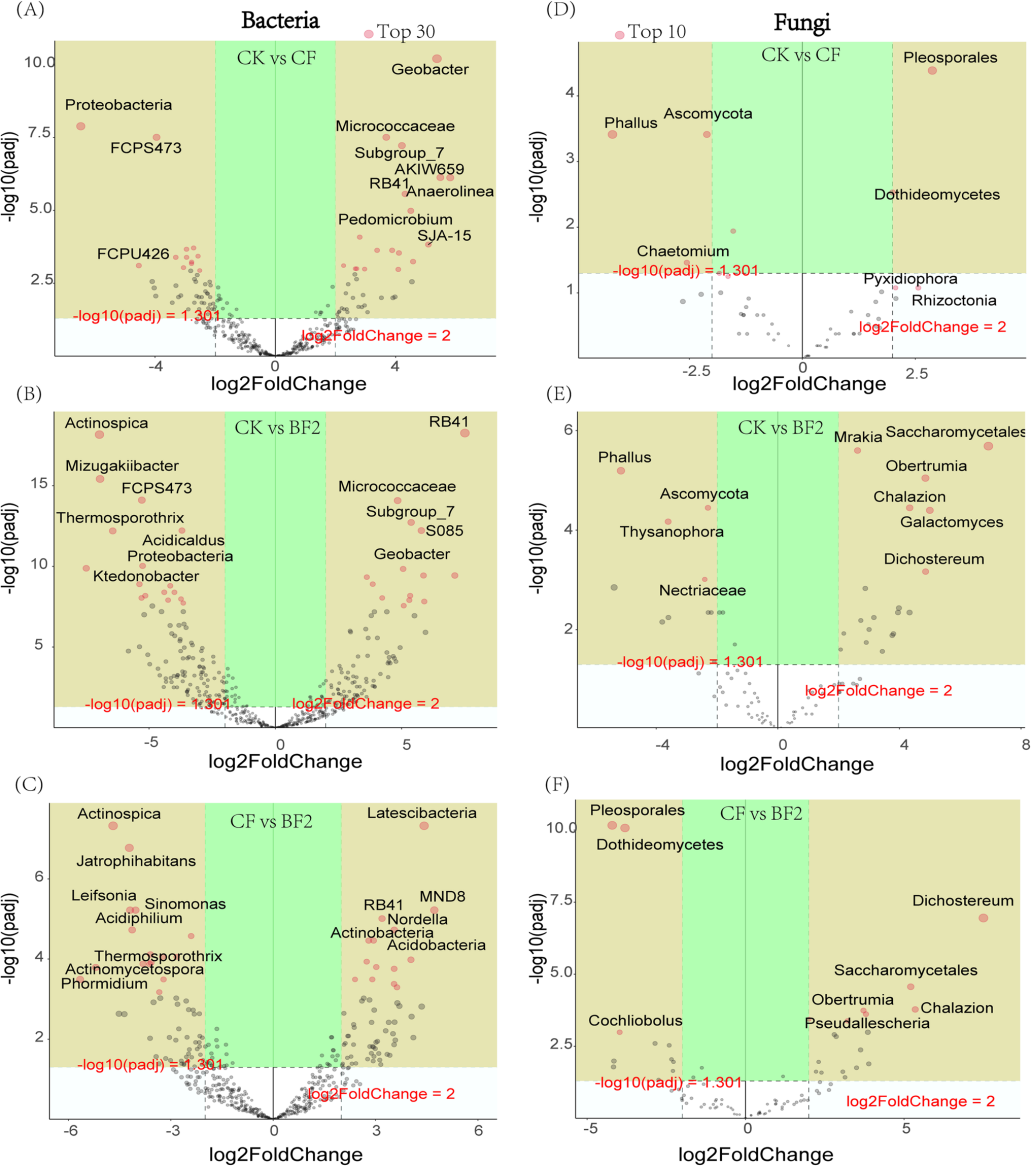
Volcano plots depicting bacteria (**A**-**C**) and fungi (**D**-**F**) genus. The *X* coordinate was |log2 (fold change)| and the *Y* coordinate was − log 10 (*p* adj), *P* < 0.05, log2 (fold change) > 2. Each point represented a genus. Points in the brown area are regulatory genera with significant changes and markers for dominant genera. Other dots were genus of non-significant difference

According to the results of DESeq2, we identified 220 genus including 98 upregulated genus and 122 downregulated genus after the comparison between CK and BF2 in the bacteria, 86 genus (up = 40, down = 46) between CK and CF, and 29 genus (up = 19, down = 10) between CF and BF2, respectively (Table S4). *Latescibacteria*, *Actinobacteria*, *Acidobacteria*, and *Nordella* were significantly enriched in comparison of CF and BF2; however, *Actinospica*, *Jatrophihabitans*, *Leifsonia*, and *Sinomonas* were significantly reduced (Fig. 5C). In the fungal community, 4 (CK vs. CF), 29 (CK vs. BF2), and 28 (CF vs. BF2) differential genera were identified in the comparison groups of the different treatments, respectively (Fig. 5D-F). *Mrakia*, *Saccharomycetales*, *Obertrumia*, and *Galactomyces* were significantly enriched after BF2 treatment compared to the control group, and *Phallus*, *Ascomycota*, and *Thysanophora* were significantly reduced (Fig. 5E). The identified differentially genus were shown by volcano plot (Fig. 5). In the volcano plot, *p* < 0.05 was set as the cut-off criterion of significant difference.

### Effects of Fertilizer Treatments on Rhizosphere Microbial Biomarkers and Functions

**Fig. 6:**
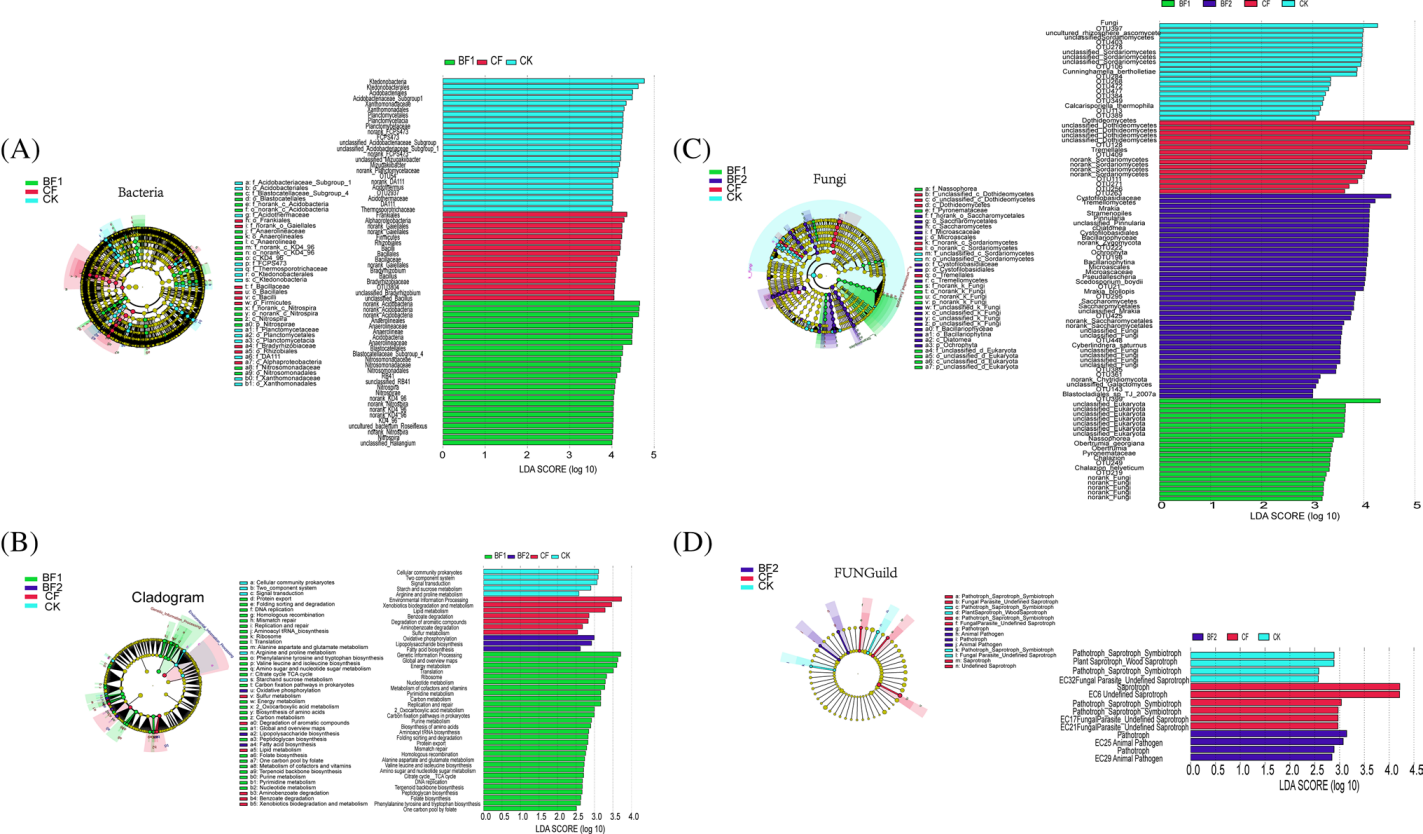
Cladogram illustrating the phylogenetic dynamics of the rhizosphere microorganisms associated with different fertilizes (**A** and **C**). Bacterial biomarkers with LDA scores of ≥ 3.5 in each treatment were listed and the LDA scores of fungi ≥ 3. Different colors depict different treatments while circles show phylogenetic levels from phylum to OTU. KEGG functional pathways are differentially abundant by different fertilizes. Differentially abundant KEGG functional pathways in sugarcane’s PICRUSt predicted metagenome and differences in functional classification of fungi FUNGuild were shown by using LEfSe (**B** and **D**). The nodes of different colors represent the microbes that perform a crucial role in the grouping illustrated in the color, and yellow nodes denote non-significant

Linear discriminant effect size (LEfSe) analysis was conducted to identify and select unique microbial taxa significantly related to each fertilizer treatment. Biomarker bacterial and fungal community were depicted in cladograms, and bacterial linear discriminant analysis (LDA) scores ≥ 3.5 and fungal LDA ≥ 3 were then performed respectively (Fig. 6A and C). Biomarkers associated with treatments varied across the fertilizer. The bacterial and fungal community LDA analysis detected 66 (CK = 24, CF = 16, BF1 = 26, BF2 = 0) and 98 (CK = 20, CF = 15, BF1 = 21, BF2 = 42) biomarkers for different fertilizers respectively (Fig. 6A and C). The higher score biomarker bacterial of BF1 treatment belonged to phyla *Acidobacteria* and *Anaerolineaceae*; that of CF belonged to *Alphaproteobacteria*, *Gaiellales*, and *Frankiales*. Meanwhile, in fungi, the higher score biomarker of BF2 belonged to *Cystofilobasidiaceae*, *Mrakia*, *Pinnularia*, and *Tremellomycetes*; that of CF belonged to unclassified *Dothideomycetes* and *Tremellales* (Fig. 6C). In addition, regarding KEGG, 44 pathways were significantly different in third-level pathways (LDA > 2.5, *P* < 0.05, Fig. 6B), including 29 pathways with significant difference in BF1, such as genetic information processing, global and overview maps, and energy metabolism. Seven pathways were significantly different in CF, such as environmental information processing, lipid metabolism, and xenobiotic biodegradation and metabolism (Fig. S4). The BF1 treatment group had the most differential pathways. Meanwhile, there were 14 fungal FUNGuild (CK = 4, CF = 6, BF1 = 0, BF2 = 4), of which BF2 mainly included pathotroph and animal pathogen, and pathotroph-saprotroph and fungal parasite-undefined saprotroph were in CF (LDA > 2.0, *P* < 0.05, Fig. 6D and Fig. S5).

**Fig. 7:**
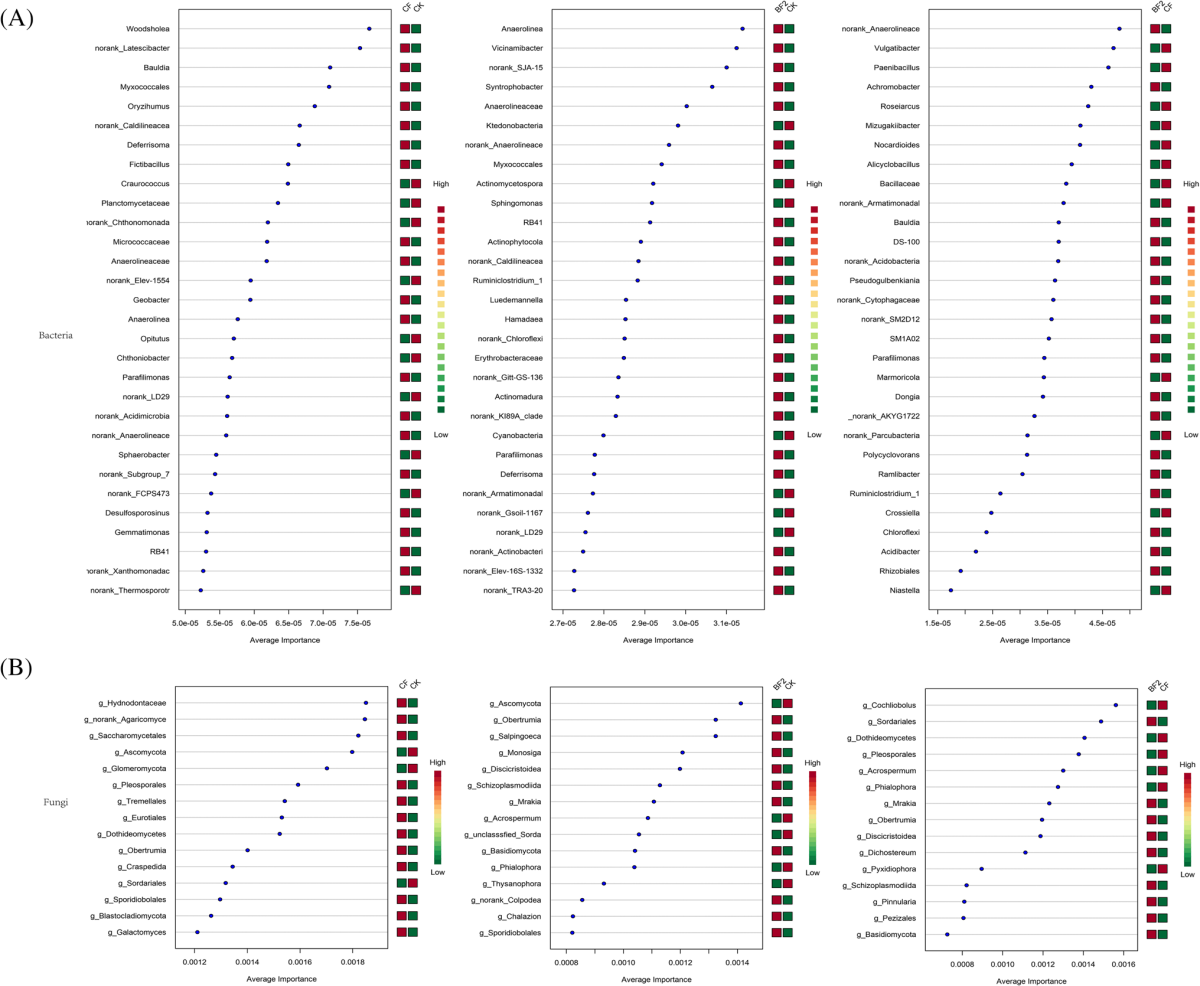
A support vector machine (SVM) approach was used to select the bacterial genera (**A**) and fungal genera (**B**) with the highest contribution to the variance in the different fertilizer treatment groups. The horizontal coordinate is the average importance and the vertical coordinate is the microbial genus, and the heatmap shows the relative abundance differences between microbial genera between the two comparison groups. Bacteria showed the top 30 genera in importance and fungi showed the top 15 genera. Order of comparison: CK vs. CF, CK vs. BF2, and CF vs. BF2

In the bacteria, of the top 30 genera identified by a support vector machine (Fig. S3), *Woodsholea*, *norank_Latescibacter*, *Bauldia*, *Myxococcales*, and *Oryzihumus* were all identified as important variables that significantly contributed to the class separation between CK and CF, *Anaerolinea*, *Vicinamibacter*, *Syntrophobacter*, and *Anaerolineaceae* were the more important genera for the difference between CK and BF2, and more attention needs to be paid to the more important role of *norank_ Anaerolineace*, *Vulgatibacter*, *Paenibacillus*, *Achromobacter*, and *Roseiarcus* for their differentiation between CF and BF2 (Fig. 7A). On the other hand, in the fungi, *Hydnodontaceae*, *norank_ Agaricomyce*, *Saccharomycetales*, *Ascomycota*, and *Glomeromycota* between CK and CF, *Ascomycota*, *Obertrumia*, *Salpingoeca*, *Monosiga*, and *Discicristoidea* between CK and BF2, and *Cochliobolus*, *Sordariales*, *Dothideomycetes*, *Pleosporales*, and *Acrospermum* between CF and BF2 had a greater contribution to the variability between groupings than other genus, respectively (Fig. 7B).

### Network Analysis of Soil Microbial Communities (Co-occurrence Network)

**Fig. 8:**
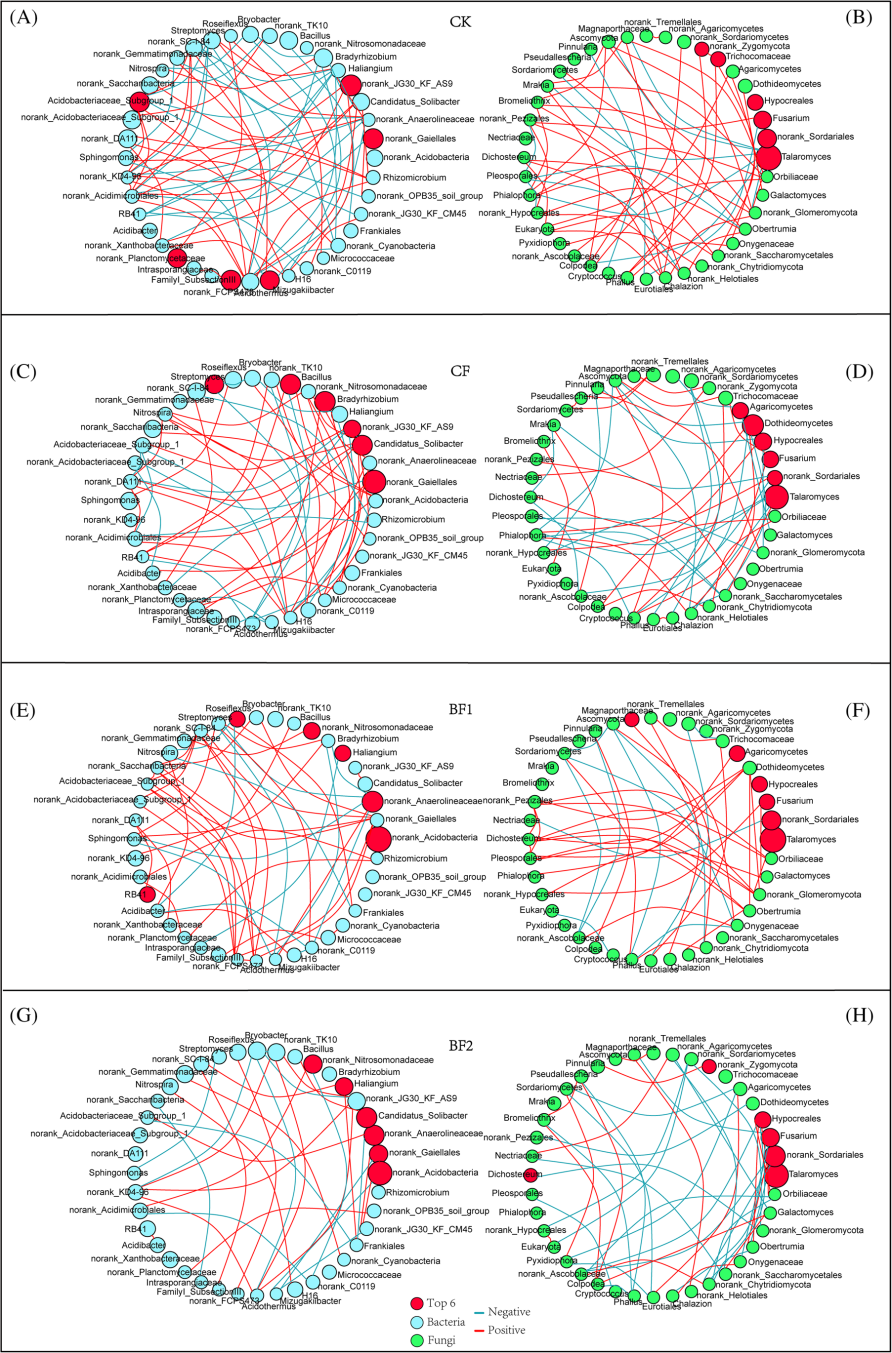
Co-occurrence networks of rhizosphere microbial features. The map shows the bacterial and fungal networks at the genus level, respectively, and then showed the bacterial and fungal networks with top 40 genera, respectively. CK: urea application (**A** and **B**), CF: compound fertilizer (**C** and **D**), BF1: bio-fertilizer + urea (**E** and **F**), and BF2: bio-fertilizer + urea (**G** and **H**). Different lines represent two significant Pearson correlations (*r*^2^ > 0.4, *p* < 0.05). Light red lines represent a significant positive correlation and blue lines represent a significant negative correlation. The red nodes represent the top 6 node values in each network, and the size of the circle represents the relative abundance of each genera

Co-occurrence network analysis was used to assess interactions across dominant populations, and only the significant correlations (*r*^2^ > 0.4, *p* < 0.05) were shown in this network. The results revealed a lower number of links in the BF2 in the bacteria, and in the fungi, BF1 feature networks had the least number of links (Table S5). Further insight into the bacterial and fungal genera network illustrated the lowest mean degree, centralization closeness, network centralization, and clustering coefficient values in BF2 than other treatments (Table S6). Some genus, such as *norank_Acidobacteria*, *norank_Anaerolineaceae*, *Bacillus*, and *Roseiflexus*, had a higher relative abundance and clustering coefficient in the bacterial network of BF1. The genus *Candidatus_Solibacter*, *norank_Nitrosomonadaceae*, *Nitrospira*, and *norank_Acidimicrobiales* of CF in bacterial network had the largest clustering coefficient compared with other treatments (Fig. 8C and Table S7). In fungal network, *Fusarium* had the highest clustering coefficient values in CF compared to other treatments; however, BF2 had the lowest clustering coefficient value (Fig. 8D and F, Table S8).

## Discussion

Fertilizer application is one of the most common agricultural practices used in agricultural production activities to increase crop yields [38, 39]. Although the nutrient use efficiency in China’s farming activities has gradually improved over the past decade [40], a large amount of inorganic fertilizers (nitrogen, phosphorous, and potassium) have been applied to farmland in order to increase crop yields, which has caused many serious ecological problems, such as soil organic matter loss [41], low soil fertility, nutrient inefficiency, and soil quality degradation [43]. In this dangerous environment where the intake of chemical fertilizers cannot continue to improve yields, the development of new fertilizers is a very important milestone. At the same time, an in-depth understanding of the activity pattern of rhizosphere soil microorganisms after bio-fertilizer application can play a crucial role in the better development and utilization of new fertilizers to improve soil productivity. Therefore, we conducted this study.

### Impact of Different Fertilizers on Sugarcane Yield Index and Soil Nutrients

Until now, there is enough evidence that soil physicochemical factors such as SOC, TP, TN, AP, AN, and AK are enhanced by different fertilizers; at the same time, some fertilizers can mitigate soil acidification to some extent [43] [41]. However, these studies are based on chemical fertilizers or other fertilizers, and rhizosphere microbial studies related to bio-fertilizers are still relatively lacking. Our findings suggest that sugarcane sugar and soil pH showed noticeable variation among different fertilizers, which may be attributable to the fact that the microorganisms added to the bio-fertilizer promote the increase of sugarcane root secretion or the rhizosphere community under the bio-fertilizer recruits more functional microbes from the soil that facilitate soil acidity reduction and nutrient uptake by the roots [13]. Although the addition of bio-fertilizer did not result in a significant level of difference in yield indicators compared to the CF treatment group, the yield increase with the use of bio-fertilizer was greater than the addition of chemical fertilizers. Similarly, the input of organic matter in the bio-fertilizer can improve the water-soluble and exchangeable forms of soil micronutrients, further enhancing the uptake of soil micronutrients by the sugarcane root system [45].

### Effect of Fertilizers on Microbial Species Composition and Diversity

Fertilizer addition significantly affected the diversity and species composition of the sugarcane rhizosphere microbial community. The results showed that both compound fertilizer and bio-fertilizer increased bacterial diversity and abundance to different degrees, but had no significant effect on the rhizosphere fungal community. This phenomenon is similar to the findings of Bello et al. [46]. The non-metric multidimensional scaling (NMDS) and redundancy analysis (RDA) were used to explore changes in the composition of the rhizosphere microbial community and the correlation between environmental factors and the rhizosphere community, respectively. The results indicated that samples from different treatment groups in NMDS (Fig. 4A and D) were significantly separated and then clustered together, and the Adonis test (Table 3) once again proved that there was a significant difference between the fertilizer treatments (*p* < 0.05). Many studies have demonstrated that soil physicochemical factors are important drivers of soil microbial communities [47, 48]. Likewise, our finding revealed that pH, AN, TN, AK, and SOC significantly affected the rhizosphere bacterial and fungal structure and diversity according to RDA and Spearman correlation heatmap analyses (Fig. 3). The results of the VPA analysis likewise revealed that soil physicochemical variables explained a large proportion of the microbial variation (Fig. S2). These results support some of the previous findings, Cao et al. who reported that soil pH, SOC, TN, and TP were all significantly correlated with bacteria, fungi, and total microorganisms [49]. These observations may be due to the fact that the properties of different fertilizers can have specific effects on rhizosphere environment, and that functional bacteria in bio-fertilizers may increase the availability of nutrients or promote the secretion of certain chemicals from sugarcane while influencing rhizosphere community interactions, thus affecting the entire root-soil-microbial system. In addition, the bacterial genera that showed significant positive correlation with TN, TP, and AK in this study were *Acidimicrobiales*, *Haliangium*, *Nitrospira*, and *Nitrosomonadaceae*; and the major fungal genera were *Pseudallescheria*, *Mrakia*, *Chalazion*, and *Chytridiomycota.* These microbial genera are likely to act as coordinators or transformers of nutrients in the soil [50, 51].

### Fertilizer’s Effect on Differential Microbes

There was a large variability of differential microbial genus in comparison groups (Fig. 5 and Table S4). The bacterial genera *Microbacterium*, *Leifsonia*, and *Sinomonas* that were significantly reduced in BF2 compared to CK and CF were reported as a group of gram-positive bacteria may associated with disease [52]; in particular, the reduction of *Leifsonia* is likely to suppress or slow down the occurrence of ratoon stunting (growth-hindering) disease of sugarcane [53]. Meanwhile, significantly enriched *Geobacter*, *Nitrosomonadaceae*, and *Pedomicrobium* were associated with environmental remediation [54], nitrification, and utilization of trace elements in the soil [55, 56], and microbial interactions may have promoted the activity of rhizosphere-related enzymes in sugarcane, thus facilitating the uptake and utilization of trace elements. In addition, in the fungal volcano map (Fig. 5B), compared with CK and CF treatment groups, the increase of *Saccharomycetales* could synthesize the active chemical substances that promote root growth and cell division and promote the substrate required for the proliferation of other effective microorganisms [57]. The emergence of these phenomenons has deepened our understanding of the role of bio-fertilizers in promoting soil ecosystems and plant health in several ways.

### Impact of Fertilizers on Biomarkers and Functions

To further explore the effects brought by the bio-fertilizer on the rhizosphere community, LEfSe analysis and machine algorithm (support vector machine, SVM) were used to find biomarkers and the differential contribution of microbial genera in different treatment groups, respectively. According to the results of LEfSe analysis, microbial indicator differs significantly among fertilizer treatments. This suggested that the treatment with different fertilizers accelerated the selection of the rhizosphere microbial community by modifying the rhizosphere soil microenvironment and releasing chemical secretions (recruitment or expulsion) by sugarcane to build a suitable rhizosphere environment for its own growth [58, 59]. Most of all significant biomarkers belong to *Acidobacteria*, *Actinobacteria*, and *Proteobacteria* in bacterial groups and *Ascomycota* and *Basidiomgcota* in fungi community. Such results once again corroborated the observation of Zhang et al., who reported phylum *Ascomycota* to be the most pronounced biomarker microbial community under different carbon assimilation [60]. Meanwhile, SVM evaluated the importance of the microbial genera responsible for the variability between fertilizers. Microbial genera of relatively high average importance may influence functional differences in sugarcane under fertilizer measures [61]. Between BF2 and CF, the top ranked bacterial genera in terms of relative importance were *Anaerolineace*, *Vulgatibacter*, and *Paenibacillus* and fungi were *Cochliobolus*, *Sordariales*, and *Dothideomycetes.* Microbial genera of high importance may be associated with biological processes significantly marked in LEfSe (Fig. 6B and D). Furthermore, among the LEfSe of bacterial functional pathway, BF1 had the most tagged functional pathways, such as genetic information processing, global and overview maps, energy metabolism, translation, citrate cycle, and TCA cycle, which suggested that the addition of bio-fertilizers may affect numerous biological processes by altering the community structure and composition of rhizosphere microorganisms. In a previous study, the application of *Trichoderma* bio-fertilizer reported by Zhang et al. changed the microbial environment of the grassland and *Trichoderma* abundance became the most important contributor to the grassland biomass, suggesting from the side that the addition of bio-fertilizer changed a series of biological processes at the rhizosphere level [13], while in fungi, CF treatment seemed to have a stronger effect on the biological processes of rhizosphere fungi, and this phenomenon may be due to the contest between fertilizer effect and microbial effect, which needs to be explored more deeply [46].

### Fertilizer’s Effects on Soil Microbial Communities and Network Patterns

Co-occurrence analysis showed that the relative abundance of bacteria *Acidobacteria* and *Anaerolineaceae* was significantly higher with the addition of bio-fertilizer to the soil compared to CK and CF treatment groups (Table S9) and played a more important role in the network (Table S7). We hypothesized that the increase in abundance was closely related to the increase in rhizosphere soil pH of sugarcane. Soil pH has been reported to be one of the major soil factors determining microbial community structure under controlled conditions of different fertilizers [46, 62]. Some microorganisms can inhibit most enzyme metabolism through internal acidification of cells, and are sensitive to pH changes [63]. Thus, an increase in soil pH is in part suggestive of a healthier soil environment. We also identified some potential beneficial bacteria among the microbes with higher relative abundance and position in the BF1 and BF2 co-occurrence network; for instance, *Nitrosomonadaceae* has been reported to be closely associated with nitrification in soil and bioremediation of toxic chemicals in soil [64–66]. In addition, the network centralization of bacterial networks differed among fertilizer treatments, with BF2 having the smallest network centralization (15.52%) (Table S6), which may be due to the fact that the addition of functional bacteria in the fertilizer disrupted the equilibrium of the interaction between the original microorganisms in the soil, making the network more extensive and more key microbes become the central radiation point. In the fungal network, *Talaromyces* had absolute numerical and positional dominance in each treatment (Tables S8 and S10). However, the addition of different fertilizers resulted in more negative relationships among the genera, and the greatest increase in the rate of negative relationships was observed in the BF2 network (Table S5). Meanwhile, the fungal network with bio-fertilizer treatment possessed fewer interactions, which was similar to the network characteristics of healthy soil proposed by Yun et al. [67]. Interestingly, among the fungal networks, CF possessed the highest network centralization, which may be due to the specific effects of chemical fertilizers on fungi.

## Conclusion

In this study, we determined the rhizosphere microbial community composition, function, and response to changes in soil physicochemical parameters in sugarcane after application of different fertilizers. The main reason for such changes could be due to the combined effect of soil pH, nutrients in fertilizers, and functional bacteria. The VPA analysis showed a high degree of explanation for the microbial community by soil physicochemical factors. Compared with CK and CF, using bio-fertilizer greatly reduced soil acidification and improved soil microbial community composition and structure, thus improving soil quality and soil productivity. In addition, using bio-fertilizers induced more beneficial microorganisms to accumulate in the rhizosphere soil of sugarcane; meanwhile, the reduction of some pathogenic bacteria such as *Leifsonia* likely inhibited or slowed down the occurrence of sugarcane-persistent dwarf disease, promoting plant health. In the co-occurrence networks under different fertilizer measures, bio-fertilizer network is closer to the network characteristics of healthy soil, which indicated that the application of bio-fertilizer can improve the health of soil to some extent and achieve green and stable sustainable development. Overall, this study provides new insights into the future replacement of overused chemical fertilizers by bio-fertilizers and is important for exploring the plant-soil-microbial interactions.

## Supplementary Information

Below is the link to the electronic supplementary material.

Supplementary file1 (DOCX 2013 kb)
