# Supplementary material for: Bio-fertilizer Affects Structural Dynamics, Function, and Network Patterns of the Sugarcane Rhizospheric Microbiota
Source: Microb Ecol. 2021 Nov 24;84(4):1195–211. doi: 10.1007/s00248-021-01932-3 (PMC9747866; doi:10.1007/s00248-021-01932-3)
Supplement: Supplementary file 1 — Supplementary file1 (DOCX 2013 kb) [file 248_2021_1932_MOESM1_ESM.docx]

Table S1 Statistical table of α-diversity index under different fertilizer treatments

Note: Different letters in each column indicate significant difference among the treatments at 0.05 level.

|  | Bacteria | | | | Fungi | | | |
| --- | --- | --- | --- | --- | --- | --- | --- | --- |
|  | CK | CF | BF1 | BF2 | CK | CF | BF1 | BF2 |
| Sobs | 2069±91.66b | 2575.67±52.33a | 2441.33±93.4a | 2704.67±52a | 179.67±8.41b | 200±3.21ab | 238±14.15a | 237.67±4.10a |
| Shannon | 6.17±0.09b | 6.47±0.1ab | 6.58±0.04a | 6.73±0.01a | 3.15±0.06a | 3.19±0.04a | 3.20±0.14a | 3.34±0.05a |
| Ace | 2527.86±122.61b | 3214.07±54.19a | 2942.86±126.57ab | 3275.72±38.83a | 209.02±13.07b | 232.92±13.28ab | 262.96±15.48ab | 269.01±9.19a |
| Chao | 2533.87±104.74b | 3215.22±60.74a | 3020.17±166.33a | 3334.88±34.64a | 238.34±32.42a | 228.80±10.96a | 263.55±17.81a | 274.99±15.09a |


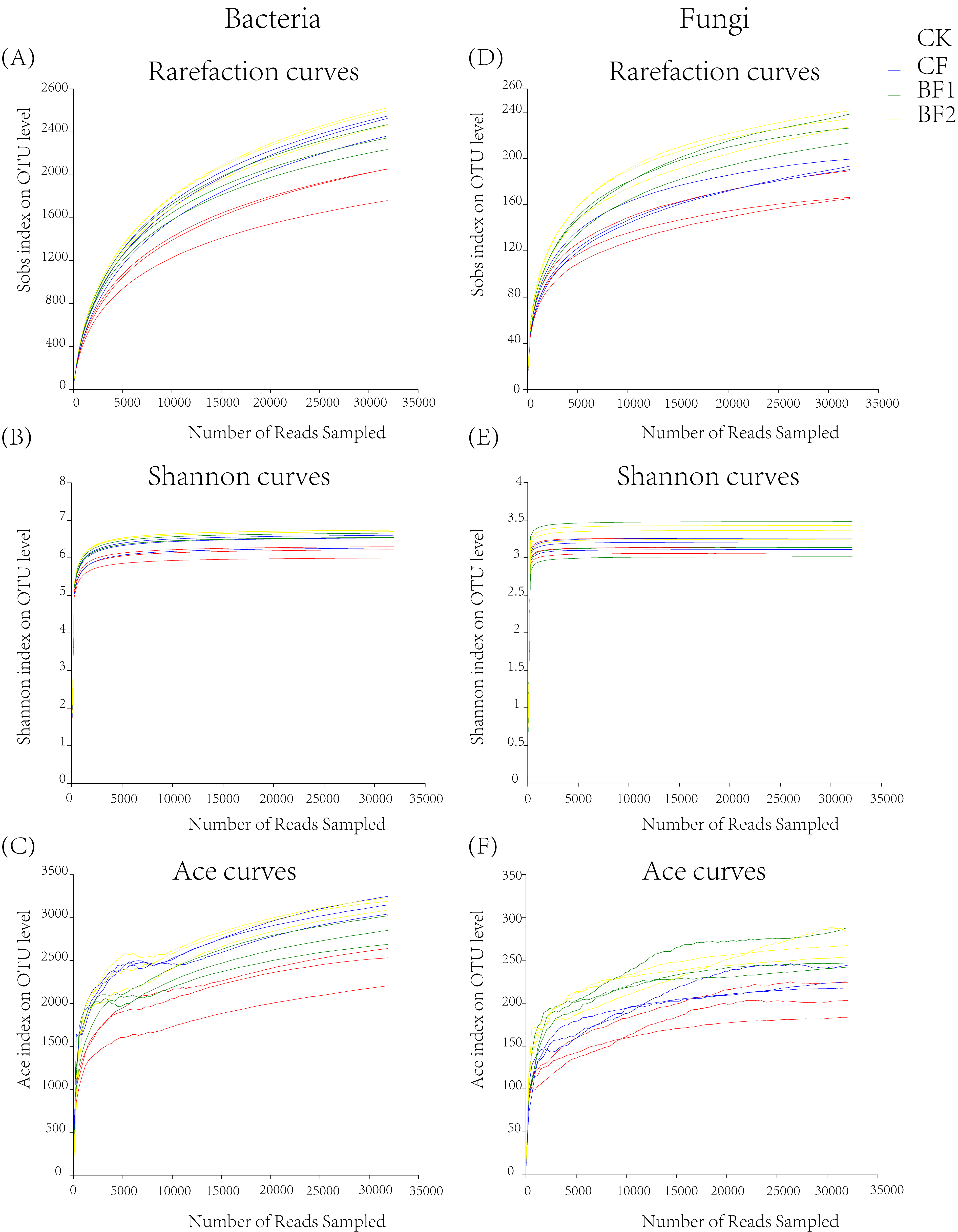


Fig.S1. The rarefection curve of bacterial α-diversity index (A-C), and the rarefection curve of the fungal related index (D-F). Different colors in the picture represent different fertilization treatments. CK: urea application (57 kg/ha), CF: compound fertilizer (450 kg/ha, BF1: bio-fertilizer (1,500 kg/ha of bio-fertilizer + 57 kg/ha of urea), BF2: bio-fertilizer (2250 kg/ha of bio-fertilizer + 57 kg/ha of urea).

Table S2 Summary of the percentages of the top 10 microbial phyla in samples treated with different fertilizers

| **Bacteria/**Fungi phyla | **CK-1** | **CK-2** | **CK-3** | **CF-1** | **CF-2** | **CF-3** | **BF1-1** | **BF1-2** | **BF1-3** | **BF2-1** | **BF2-2** | **BF2-3** |
| --- | --- | --- | --- | --- | --- | --- | --- | --- | --- | --- | --- | --- |
| **Proteobacteria** | 0.2526 | 0.302 | 0.3391 | 0.3604 | 0.2946 | 0.3389 | 0.2565 | 0.3293 | 0.304 | 0.2914 | 0.3455 | 0.3816 |
| **Actinobacteria** | 0.2323 | 0.2823 | 0.1548 | 0.3108 | 0.3291 | 0.2195 | 0.1923 | 0.1278 | 0.1656 | 0.1821 | 0.157 | 0.1482 |
| **Chloroflexi** | 0.1991 | 0.1827 | 0.1818 | 0.0976 | 0.1318 | 0.1638 | 0.1922 | 0.2079 | 0.2139 | 0.2171 | 0.1796 | 0.161 |
| **Acidobacteria** | 0.1372 | 0.0687 | 0.181 | 0.0976 | 0.0869 | 0.1298 | 0.1733 | 0.1948 | 0.1953 | 0.205 | 0.2037 | 0.1735 |
| **Firmicutes** | 0.0448 | 0.0579 | 0.0616 | 0.0575 | 0.0546 | 0.0668 | 0.0211 | 0.0188 | 0.0254 | 0.0298 | 0.0327 | 0.026 |
| **Cyanobacteria** | 0.0437 | 0.0195 | 0.0048 | 0.0075 | 0.0542 | 0.0109 | 0.1014 | 0.0221 | 0.0209 | 0.0028 | 0.0045 | 0.0188 |
| **Gemmatimonadetes** | 0.0167 | 0.0148 | 0.0376 | 0.0277 | 0.0123 | 0.0222 | 0.0184 | 0.0281 | 0.0256 | 0.0334 | 0.0332 | 0.0407 |
| **Planctomycetes** | 0.0576 | 0.0475 | 0.029 | 0.0071 | 0.0143 | 0.0119 | 0.0065 | 0.0063 | 0.0121 | 0.0116 | 0.0089 | 0.0057 |
| **Bacteroidetes** | 0.0126 | 0.0208 | 0.0084 | 0.0198 | 0.0196 | 0.0269 | 0.0276 | 0.0184 | 0.0159 | 0.0108 | 0.0145 | 0.0208 |
| **Nitrospirae** | 0.0034 | 0.0038 | 0.0019 | 0.0141 | 0.0025 | 0.0093 | 0.0108 | 0.0466 | 0.0213 | 0.0161 | 0.0204 | 0.0238 |
| Ascomycota | 0.8749 | 0.9173 | 0.8313 | 0.8094 | 0.8206 | 0.8046 | 0.7472 | 0.8496 | 0.8202 | 0.8074 | 0.7939 | 0.7321 |
| Basidiomycota | 0.0768 | 0.0432 | 0.0743 | 0.1642 | 0.1519 | 0.1607 | 0.2004 | 0.0856 | 0.1063 | 0.0951 | 0.1565 | 0.1254 |
| Zygomycota | 0.0224 | 0.0284 | 0.085 | 0.014 | 0.0157 | 0.0135 | 0.0134 | 0.0289 | 0.0237 | 0.0322 | 0.0202 | 0.0616 |
| Ciliophora | 0.0148 | 0.0049 | 0.0033 | 0.0066 | 0.0082 | 0.0123 | 0.0213 | 0.0206 | 0.0252 | 0.0313 | 0.0149 | 0.0137 |
| Ochrophyta | 0.0042 | 0.0041 | 0.0039 | 0.0034 | 0.0021 | 0.0034 | 0.0089 | 0.0043 | 0.0205 | 0.0223 | 0.0073 | 0.0539 |
| Chytridiomycota | 0.002 | 0.0006 | 0.001 | 0.0009 | 0.0007 | 0.0026 | 0.0013 | 0.0013 | 0.002 | 0.0042 | 0.0022 | 0.0044 |
| Choanomonada | 0.0008 | 0.0004 | 0.0005 | 0.0006 | 0.0005 | 0.0019 | 0.0009 | 0.0067 | 0.0006 | 0.0025 | 0.0038 | 0.0029 |
| Glomeromycota | 0.004 | 0.0011 | 0.0005 | 0.0004 | 0.0003 | 0.0004 | 0.004 | 0.0003 | 0.0004 | 0.0038 | 0.0001 | 0.0021 |
| Schizoplasmodiida | 0.0001 | 0 | 0.0001 | 0.0002 | 0 | 0.0002 | 0.0019 | 0.0022 | 0.0004 | 0.0007 | 0.001 | 0.0021 |
| Blastocladiomycota | 0.0001 | 0.0001 | 0 | 0.0002 | 0.0001 | 0.0005 | 0.0006 | 0.0004 | 0.0007 | 0.0003 | 0.0001 | 0.0019 |

Table S3 Summary of specific values in RDA analysis

|  | Bacteria | | | | Fungi | | | |
| --- | --- | --- | --- | --- | --- | --- | --- | --- |
|  | RDA1 | RDA2 | r^2^ | p-value | RDA1 | RDA2 | r^2^ | p-value |
| pH | 0.8145 | 0.5802 | 0.8070 | 0.0005 | 0.9843 | -0.1764 | 0.664 | 0.0045 |
| SOC | 0.9897 | -0.1433 | 0.4474 | 0.0830 | 0.929 | 0.3702 | 0.6974 | 0.0025 |
| TN | 0.9989 | -0.0471 | 0.5918 | 0.0235 | 0.9556 | 0.2945 | 0.7558 | 0.0020 |
| TP | 0.8006 | -0.5993 | 0.5920 | 0.024 | 0.9968 | -0.0794 | 0.3494 | 0.1359 |
| AN | 0.9270 | -0.3750 | 0.5780 | 0.0105 | 0.9966 | 0.0826 | 0.2437 | 0.2939 |
| AK | 0.9505 | -0.3108 | 0.7988 | 0.001 | 0.8393 | -0.5437 | 0.6303 | 0.0085 |
| AP | 0.8339 | -0.5518 | 0.0368 | 0.8546 | 0.8617 | 0.5074 | 0.0280 | 0.8681 |

**
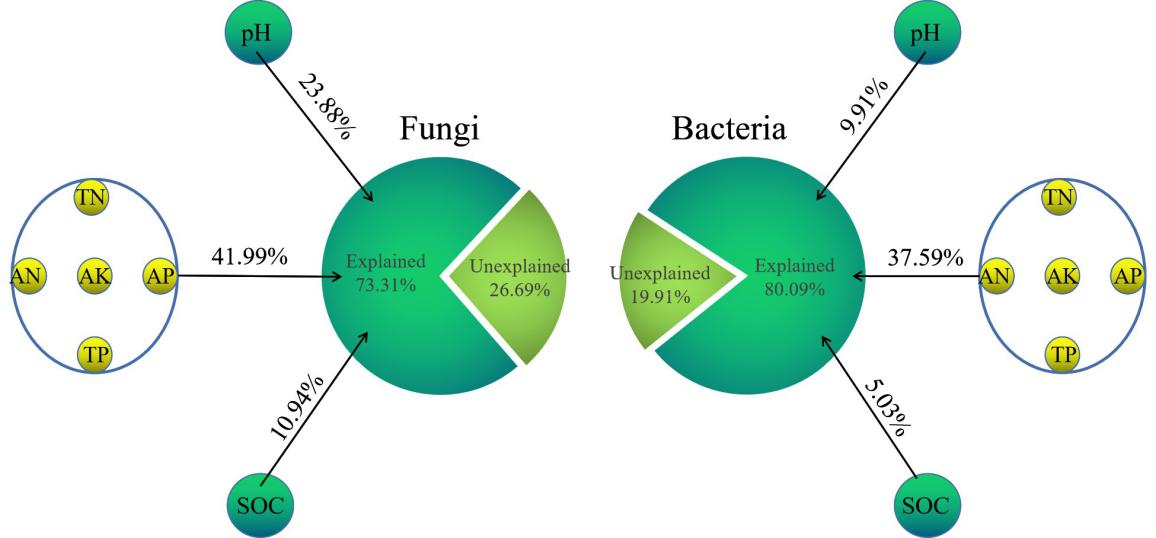
**

Fig. S2. The map quantitatively analyzed the degree of explanation of the effects of different soil environmental factors on the species changes of microbial communities in the rhizosphere of sugarcane, with fungi on the left and bacteria on the right.

Table S4 Microbial genera that changed significantly in different comparison groups

|  | Bacteria | | | Fungi | | |
| --- | --- | --- | --- | --- | --- | --- |
|  | Upregulated | Downregulated | Sum | Upregulated | Downregulated | Sum |
| CK vs CF | 40 | 46 | 86 | 1 | 3 | 4 |
| CK vs BF2 | 98 | 122 | 220 | 19 | 10 | 29 |
| CF vs BF2 | 19 | 10 | 29 | 17 | 11 | 28 |


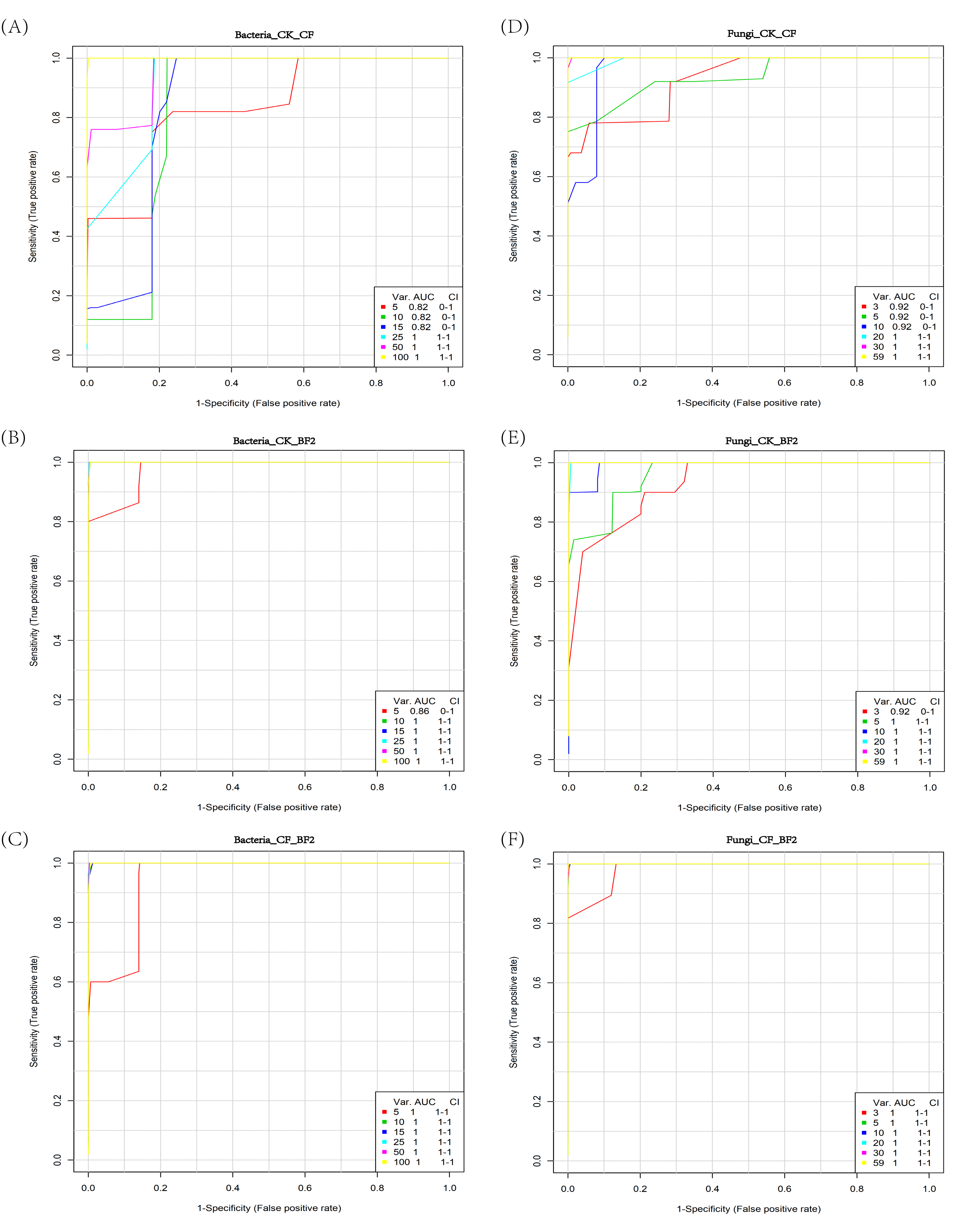


Fig. S3. The ROC curves of Support Vector Machine (SVM) for different comparison groups are false positive rate (FPR) in the horizontal coordinate and true positive rate (TPR) in the vertical coordinate. AUC represents the area under the curve. The larger the AUC value, the closer the ROC curve is to the upper left corner, and the better the classification result. Different models have different decision values (different ROC curves) for the same set of data.

Table S5 Summary of the interactions of microbial genera in the network diagram

|  | Bacteria | | | | Fungi | | | |
| --- | --- | --- | --- | --- | --- | --- | --- | --- |
|  | Positive | Negative | Sum | Positive/Negative | Positive | Negative | Sum | Positive/Negative |
| CK | 72 | 62 | 134 | 1.16 | 80 | 28 | 108 | 2.85 |
| CF | 66 | 42 | 108 | 1.57 | 48 | 48 | 96 | 1 |
| BF1 | 74 | 26 | 100 | 2.84 | 60 | 22 | 82 | 2.72 |
| BF2 | 40 | 26 | 66 | 1.53 | 38 | 54 | 92 | 0.7 |

Table S6 Global network properties of top 40 microbial genera

|  | Bacteria | | | | Fungi | | | |
| --- | --- | --- | --- | --- | --- | --- | --- | --- |
|  | CK | CF | BF1 | BF2 | CK | CF | BF1 | BF2 |
| Mean degree | 1.528 | 0.208 | 4.534 | 0.988 | 1.721 | 1.129 | 0.996 | 0.517 |
| Clustering coefficient | 0.794 | 0.783 | 0.814 | 0.740 | 0.800 | 0.775 | 0.786 | 0.744 |
| Centralization closeness | 2.442 | 2.403 | 2.505 | 2.417 | 2.431 | 2.433 | 2.426 | 2.428 |
| Network centralization | 29.00% | 22.40% | 22.40% | 15.52% | 19.43% | 21.32% | 19.97% | 17.00% |

Table S7 Summary of clustering coefficients for each bacterial genus in the network diagram

| Bacteria | Clustering coefficient | | | |
| --- | --- | --- | --- | --- |
|  | CK | CF | BF1 | BF2 |
| norank_Acidobacteria | 0.906 | 0.802 | 0.917 | 0.765 |
| norank_Gaiellales | 0.674 | 0.68 | 0.871 | 0.765 |
| norank_Anaerolineaceae | 0.787 | 0.817 | 0.864 | 0.633 |
| Candidatus_Solibacter | 0.763 | 0.921 | 0.786 | 0.765 |
| norank_JG30_KF_AS9 | 0.889 | 0.725 | 0.786 | 0.831 |
| Haliangium | 0.792 | 0.725 | 0.652 | 0.801 |
| Bradyrhizobium | 0.848 | 0.817 | 0.846 | 0.757 |
| norank_Nitrosomonadaceae | 0.616 | 0.921 | 0.802 | 0.684 |
| Bacillus | 0.538 | 0.802 | 0.846 | 0.765 |
| norank_TK10 | 0.863 | 0.802 | 0.69 | 0.831 |
| Bryobacter | 0.889 | 0.728 | 0.826 | 0.671 |
| Roseiflexus | 0.854 | 0.623 | 0.857 | 0.699 |
| Streptomyces | 0.787 | 0.725 | 0.846 | 0.85 |
| norank_SC-I-84 | 0.829 | 0.786 | 0.871 | 0.933 |
| norank_Gemmatimonadaceae | 0.889 | 0.848 | 0.762 | 0.784 |
| Nitrospira | 0.848 | 0.921 | 0.833 | 0.721 |
| norank_Saccharibacteria | 0.858 | 0.745 | 0.871 | 0.595 |
| Acidobacteriaceae_Subgroup_1 | 0.829 | 0.725 | 0.871 | 0.633 |
| norank_Acidobacteriaceae_Subgroup_1 | 0.906 | 0.61 | 0.871 | 0.684 |
| norank_DA111 | 0.889 | 0.921 | 0.667 | 0.589 |
| Sphingomonas | 0.848 | 0.623 | 0.846 | 0.667 |
| norank_KD4-96 | 0.763 | 0.779 | 0.823 | 0.831 |
| norank_Acidimicrobiales | 0.763 | 0.921 | 0.84 | 0.684 |
| RB41 | 0.59 | 0.802 | 0.667 | 0.743 |
| Acidibacter | 0.803 | 0.676 | 0.833 | 0.743 |
| norank_Xanthobacteraceae | 0.889 | 0.786 | 0.66 | 0.658 |
| norank_Planctomycetaceae | 0.854 | 0.713 | 0.84 | 0.719 |
| Intrasporangiaceae | 0.816 | 0.817 | 0.86 | 0.721 |
| FamilyI_SubsectionIII | 0.668 | 0.89 | 0.875 | 0.933 |
| norank_FCPS473 | 0.816 | 0.713 | 0.871 | 0.699 |
| Acidothermus | 0.763 | 0.752 | 0.871 | 0.831 |
| Mizugakiibacter | 0.889 | 0.733 | 0.864 | 0.674 |
| H16 | 0.889 | 0.921 | 0.833 | 0.721 |
| norank_C0119 | 0.693 | 0.728 | 0.846 | 0.649 |
| Micrococcaceae | 0.854 | 0.921 | 0.901 | 0.69 |
| norank_Cyanobacteria | 0.848 | 0.817 | 0.901 | 0.875 |
| Frankiales | 0.627 | 0.657 | 0.857 | 0.801 |
| norank_JG30_KF_CM45 | 0.693 | 0.786 | 0.803 | 0.765 |
| norank_OPB35_soil_group | 0.688 | 0.745 | 0.455 | 0.719 |
| Rhizomicrobium | 0.763 | 0.89 | 0.783 | 0.725 |

Table S8 Summary of clustering coefficients for each fungal genus in the network diagram

| Fungi | Clustering coefficient | | | |
| --- | --- | --- | --- | --- |
|  | CK | CF | BF1 | BF2 |
| Talaromyces | 0.517 | 0.747 | 0.769 | 0.701 |
| norank_Sordariales | 0.685 | 0.637 | 0.769 | 0.758 |
| Fusarium | 0.685 | 0.86 | 0.818 | 0.573 |
| Hypocreales | 0.677 | 0.9 | 0.771 | 0.758 |
| Dothideomycetes | 0.784 | 0.801 | 0.89 | 0.758 |
| Agaricomycetes | 0.83 | 0.675 | 0.704 | 0.737 |
| Trichocomaceae | 0.956 | 0.86 | 0.872 | 0.719 |
| norank_Zygomycota | 0.788 | 0.817 | 0.543 | 0.801 |
| norank_Sordariomycetes | 0.924 | 0.743 | 0.627 | 0.78 |
| norank_Agaricomycetes | 0.608 | 0.739 | 0.824 | 0.637 |
| norank_Tremellales | 0.596 | 0.881 | 0.872 | 0.824 |
| Magnaporthaceae | 0.866 | 0.817 | 0.879 | 0.784 |
| Ascomycota | 0.913 | 0.784 | 0.649 | 0.897 |
| Pinnularia | 0.656 | 0.762 | 0.872 | 0.801 |
| Pseudallescheria | 0.725 | 0.881 | 0.885 | 0.83 |
| Sordariomycetes | 0.685 | 0.881 | 0.802 | 0.661 |
| Mrakia | 0.866 | 0.59 | 0.627 | 0.869 |
| Bromeliothrix | 0.913 | 0.719 | 0.543 | 0.83 |
| norank_Pezizales | 0.846 | 0.648 | 0.89 | 0.67 |
| Nectriaceae | 0.56 | 0.817 | 0.634 | 0.637 |
| Dichostereum | 0.83 | 0.637 | 0.89 | 0.7 |
| Pleosporales | 0.623 | 0.737 | 0.89 | 0.708 |
| Phialophora | 0.913 | 0.801 | 0.704 | 0.78 |
| norank_Hypocreales | 0.846 | 0.9 | 0.871 | 0.678 |
| d__Eukaryota | 0.83 | 0.719 | 0.733 | 0.678 |
| Pyxidiophora | 0.838 | 0.756 | 0.787 | 0.661 |
| norank_Ascobolaceae | 0.846 | 0.59 | 0.623 | 0.67 |
| Colpodea | 0.866 | 0.802 | 0.813 | 0.83 |
| Cryptococcus | 0.956 | 0.881 | 0.624 | 0.69 |
| Phallus | 0.956 | 0.784 | 0.802 | 0.649 |
| Eurotiales | 0.692 | 0.881 | 0.872 | 0.737 |
| Chalazion | 0.87 | 0.881 | 0.872 | 0.801 |
| norank_Helotiales | 0.846 | 0.801 | 0.926 | 0.784 |
| norank_Chytridiomycota | 0.823 | 0.84 | 0.879 | 0.67 |
| norank_Saccharomycetales | 0.83 | 0.747 | 0.762 | 0.869 |
| Onygenaceae | 0.956 | 0.671 | 0.649 | 0.801 |
| Obertrumia | 0.846 | 0.663 | 0.89 | 0.695 |
| norank_Glomeromycota | 0.913 | 0.762 | 0.89 | 0.719 |
| Galactomyces | 0.784 | 0.802 | 0.871 | 0.869 |
| Orbiliaceae | 0.866 | 0.802 | 0.835 | 0.758 |

Table S9 Ranking of relative abundance of bacterial genera by treatment in the network diagram

|  | CK | CF | BF1 | BF2 |
| --- | --- | --- | --- | --- |
| 1 | norank_JG30_KF_AS9 | norank_Gaiellales | norank_Acidobacteria | norank_Acidobacteria |
| 2 | norank_Gaiellales | Bradyrhizobium | norank_Anaerolineaceae | Candidatus_Solibacter |
| 3 | norank_FCPS473 | Bacillus | norank_Nitrosomonadaceae | norank_Anaerolineaceae |
| 4 | Acidobacteriaceae_Subgroup_1 | Candidatus_Solibacter | Haliangium | norank_Nitrosomonadaceae |
| 5 | norank_Planctomycetaceae | Streptomyces | Roseiflexus | norank_Gaiellales |
| 6 | Mizugakiibacter | norank_JG30_KF_AS9 | RB41 | Haliangium |
| 7 | Bradyrhizobium | norank_SC-I-84 | Nitrospira | Bryobacter |
| 8 | norank_Acidobacteriaceae_Subgroup_1 | norank_Saccharibacteria | norank_KD4-96 | norank_JG30_KF_AS9 |
| 9 | norank_DA111 | Sphingomonas | norank_TK10 | norank_Gemmatimonadaceae |
| 10 | Bacillus | Intrasporangiaceae | FamilyI_SubsectionIII | norank_TK10 |

Table S10 Ranking of relative abundance of fungal genera by treatment in the network diagram

|  | CK | CF | BF1 | BF2 |
| --- | --- | --- | --- | --- |
| 1 | Talaromyces | Talaromyces | Talaromyces | Talaromyces |
| 2 | norank_Sordariales | Dothideomycetes | norank_Sordariales | norank_Sordariales |
| 3 | Fusarium | Hypocreales | Agaricomycetes | Fusarium |
| 4 | Hypocreales | Fusarium | Hypocreales | Hypocreales |
| 5 | Trichocomaceae | Agaricomycetes | Fusarium | norank_Zygomycota |
| 6 | norank_Zygomycota | norank_Sordariales | Magnaporthaceae | Dichostereum |
| 7 | Dothideomycetes | norank_Sordariomycetes | Dothideomycetes | Pinnularia |
| 8 | norank_Sordariomycetes | norank_Tremellales | norank_Sordariomycetes | Mrakia |
| 9 | Ascomycota | Trichocomaceae | norank_Agaricomycetes | Trichocomaceae |
| 10 | Agaricomycetes | norank_Agaricomycetes | Trichocomaceae | Pseudallescheria |


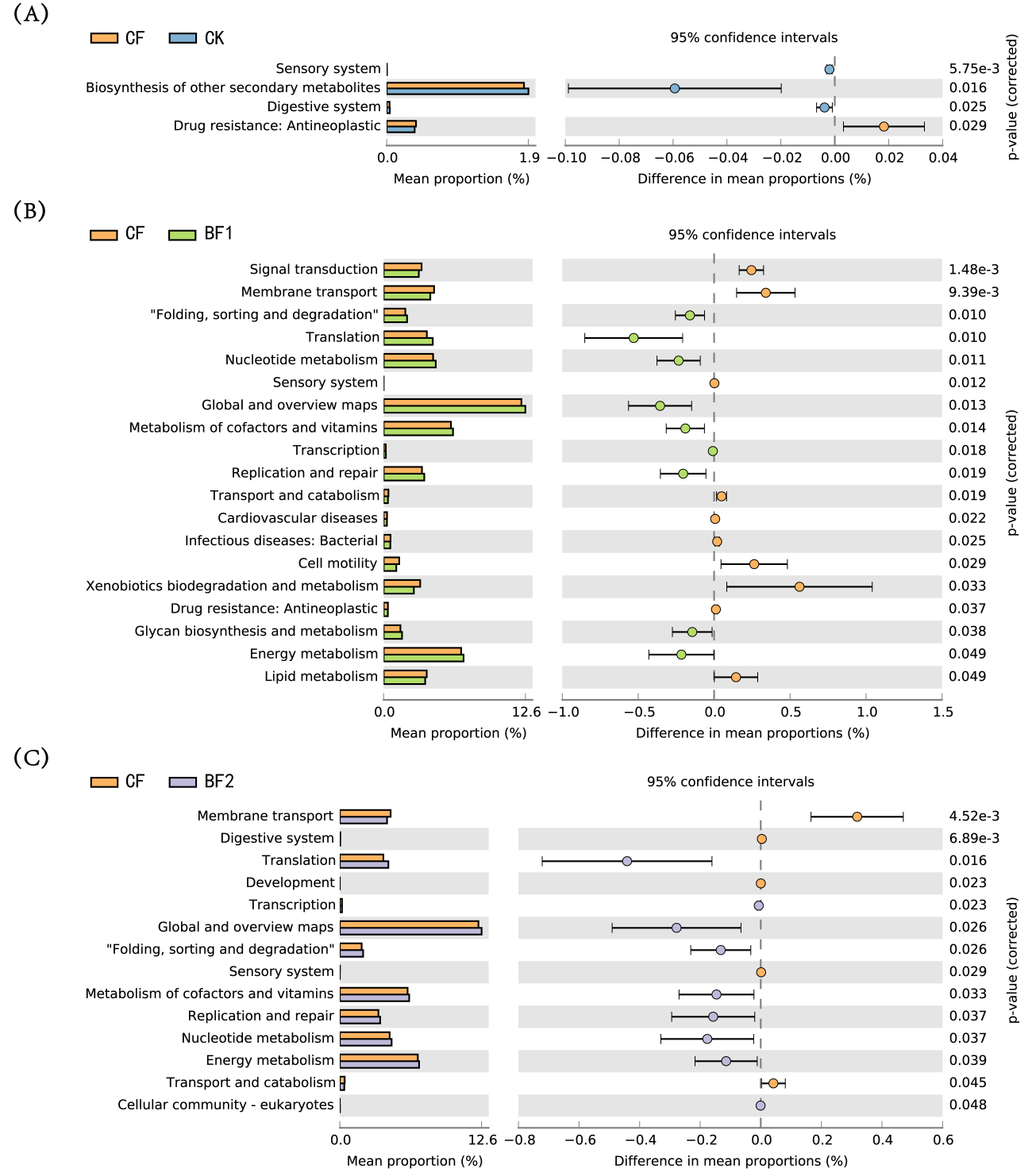


Fig. S4. Through the prediction of PICRUSt functional genes, an example of the relative abundance of significant functional differences in KEGG was obtained. The map showed the prediction results of KEGG functional pathways of soil microorganisms after different fertilization treatments, The figure illustrates the differences in CK, CF, BF1, and BF2 processing (blue, orange, green, and purple bars, respectively)


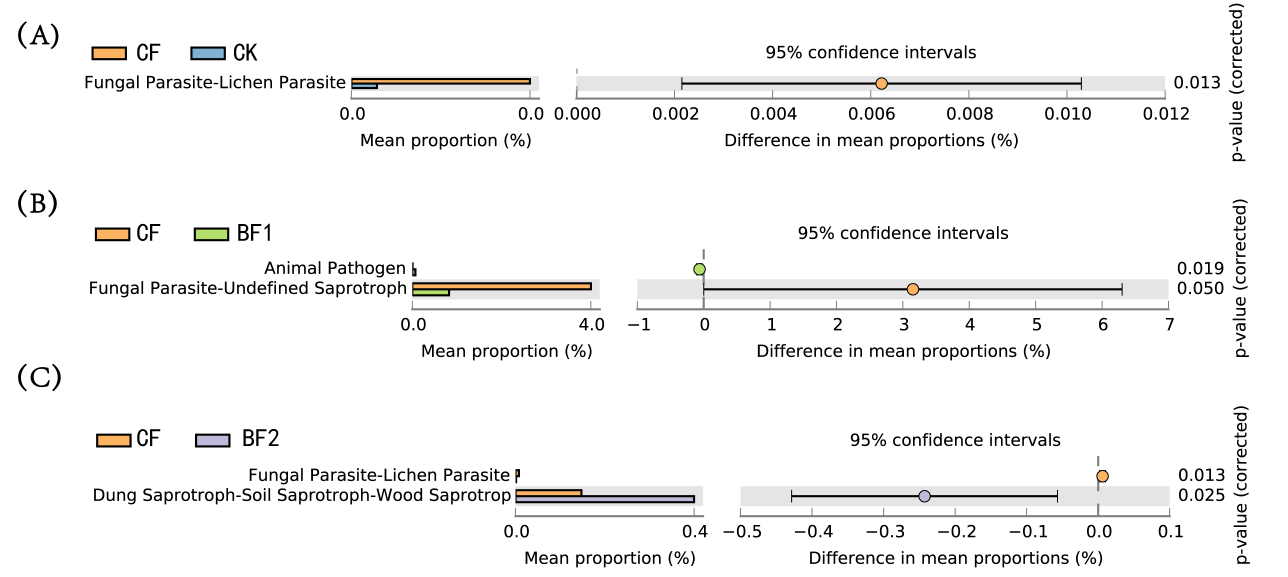


Fig. S5. Fungal function prediction analysis based on FUNGuild database, differences in CK, CF, BF1 and BF2 treatments (blue, orange, green and purple strips, respectively)
